# Project ECHO^®^: a global cross-sectional examination of implementation success

**DOI:** 10.1186/s12913-024-10920-5

**Published:** 2024-05-03

**Authors:** Perrin Moss, Nicole Hartley, Trevor Russell

**Affiliations:** 1 Integrated Care, Children’s Health Queensland Hospital and Health Service, 501 Stanley Street, 4101 QLD Australia South Brisbane,; 2 School of Health and Rehabilitation Sciences, The University of Queensland, 4072 Saint Lucia, Australia QLD; 3 School of Business, The University of Queensland, The University of Queensland, 4072 Saint Lucia, Australia QLD; 4 RECOVER Injury Research Centre, Surgical, Treatment and Rehabilitation Service (STARS), The University of Queensland, 296 Herston Rd, 4029 Australia Herston, QLD

**Keywords:** Project ECHO, ECHO, Telementoring, Implementation, Innovation, Health services research, Communities of practice, Workforce, Healthcare, Healthcare outcomes

## Abstract

**Background:**

Organizations implement innovations to disrupt the status quo and create value. Within sectors such as healthcare, innovations need to navigate large scale system and organizational factors to succeed. This research explores the implementation of a global innovation– Project ECHO^®^. Project ECHO^®^ is a validated virtual communities of practice model organizational teams implement to build workforce capacity and capability. Project ECHO^®^ has experienced broad global adoption, particularly within the healthcare sector, and is experiencing growth across other sectors. This study sought to examine the state of implementation success for Project ECHO^®^ globally, to understand how these implementations compare across geographic and sectoral contexts, and understand what enablers/barriers exist for organizational teams implementing the innovation.

**Methods:**

An empirical study was conducted to collect data on 54 Project ECHO^®^ implementation success indicators across an international sample. An online survey questionnaire was developed and distributed to all Project ECHO^®^ hub organizations globally to collect data. Data was analyzed using descriptive statistics.

**Results:**

The 54 implementation success indicators measured in this survey revealed that the adoption of Project ECHO^®^ across 13 organizations varied on a case-by-case basis, with a strong rate of adoption within the healthcare sector. Implementation teams from these organizations successfully implemented Project ECHO^®^ within 12–18 months after completing Immersion partner launch training and operated 51 ECHO^®^ Networks at the time of data collection. Implementation teams which liaised more regularly with ECHO^®^ Superhub mentors often went on to launch a higher number of ECHO^®^ Networks that were sustained over the longer term. This suggests that these implementation teams better aligned and consolidated their Project ECHO^®^ pilots as new innovations within the local context and strategic organizational priorities. Access to research and evaluation capability, and a more automated digital client relationship management system were key limitations to showcasing implementation success outcomes experienced by the majority of implementation teams.

**Conclusions:**

These findings make a valuable contribution to address a knowledge gap regarding how a global sample of organizations adopting Project ECHO^®^ measured and reported their implementation successes. Key successes included pre-launch experimentation and expansion, Superhub mentorship, stakeholder engagement, and alignment to strategic priorities.

**Supplementary Information:**

The online version contains supplementary material available at 10.1186/s12913-024-10920-5.

## Background

### Organizations implementing new innovations

Organizations implement innovations to disrupt the status quo and create new value [1–5]. Within sectors such as healthcare, implementation teams need to navigate large-scale complex systems and organizational factors to successfully embed new innovation and sustain it beyond the pilot phase [6–9]. Healthcare organizations generally experience high failure rates when implementing new digital, quality improvement and workforce development-oriented innovations [6, 10–13]. International research cites innovation failure rates between 30 and 90%, often attributed to contextual factors such as the scope of change, complexities of the specific innovation, and the absence of sustainable business models beyond pilot phase [6, 10–13]. Despite these figures, innovative practice and the adoption of new innovative models is required to advance contemporary practice and system efficiency.

The diffusion of innovation and its subsequent implementation continues to be a complex phenomenon in which executive decision-makers, academics and implementation teams in organizations grapple with [14–17]. Theories such as the Diffusion of Innovations and derivative models and frameworks cite that the rate of a new innovation’s diffusion is influenced by the time taken for diffusion to occur, how information about the innovation spreads, and the characteristics of those adopting the innovation [16, 17]. Further, five key characteristics of an innovation have been highlighted to assist with this spread– including relative advantage, compatibility, complexity, trialability, and observability [18]. Much of the published literature agrees that innovation diffusion is enabled by motivated adopters, user-friendly technology, and learner-centric training or education for adopters [13–15, 19]. Similarly, barriers to diffusion are highlighted as being poorly accessible to adopters, limitations in training and support, static policy/governance settings, limited investment streams/sustainable business models, slow speed to value and poor organizational integration [9, 20–25].

Project ECHO^®^ is an example of an innovation model with global diffusion which has been used to establish virtual communities of practice that is being increasingly adopted by organizations globally in response to strategic and operational priorities [26, 27]. The focus of this study is centered around organizations which have adopted and implemented Project ECHO^®^ as a new innovation in their efforts to establish hubs that operate virtual communities of practice.

### Communities of practice

Communities of practice have gained increasing popularity as a useful tool by which to build groups where members have a shared learning objective, and interact regularly to engage in a process of collective learning [28]. There are three key characteristics essential for a community of practice: the domain, the community, and the practice [28]. Firstly, the domain whereby the network of connections has its identity defined by its shared area of interest such as healthcare, education, climate science, quality improvement, etc. [28]. Secondly, the community where members actively participate and interact in joint activities and discussions, provide peer support and mentorship, and share information [28]. Thirdly, the practice is where members of the community are aligned in their application of and building new knowledge in a particular interest area, and subsequently growing a shared resource library of experiences, stories, tools, and solutions to resolving common problems amongst this peer group [28].

### What is Project ECHO^®^?

Project ECHO^®^ is an exemplar of a new innovation within the healthcare sector which disrupts the status quo and creates value for organizations by establishing virtual communities of practice to build workforce capacity and capability at scale [29]. Project ECHO^®^ (ECHO^®^ is an acronym for Extension for Community Healthcare Outcomes), is a licensed and trademarked hub-and-spoke telementoring model which has been implemented in over 1000 organizations globally, largely within the healthcare sector (henceforth also referred to as ECHO^®^, and the ECHO model™) [30]. The ECHO model™’s hub-and-spoke approach to building workforce development and capacity building at scale was developed using the key principles of Social Cognitive Theory, Situated Learning Theory, and Community of Practice Theory [31].

Since it was first piloted by the ECHO Institute™ (the licensor) at the University of New Mexico in the United States during 2002, the model’s use has been widely validated with over 560 peer-reviewed publications exploring various global applications of the model as it has continued to diffuse [26, 30]. This growing evidence base has spurred the adoption of the innovation by new organizations, with a particular increase observed since the COVID-19 pandemic whereby organizations needed to quickly reorientate portions of their business operations to become virtual in order to sustain and support workforce development and capacity building activities [1, 30].

**Fig. 1:**
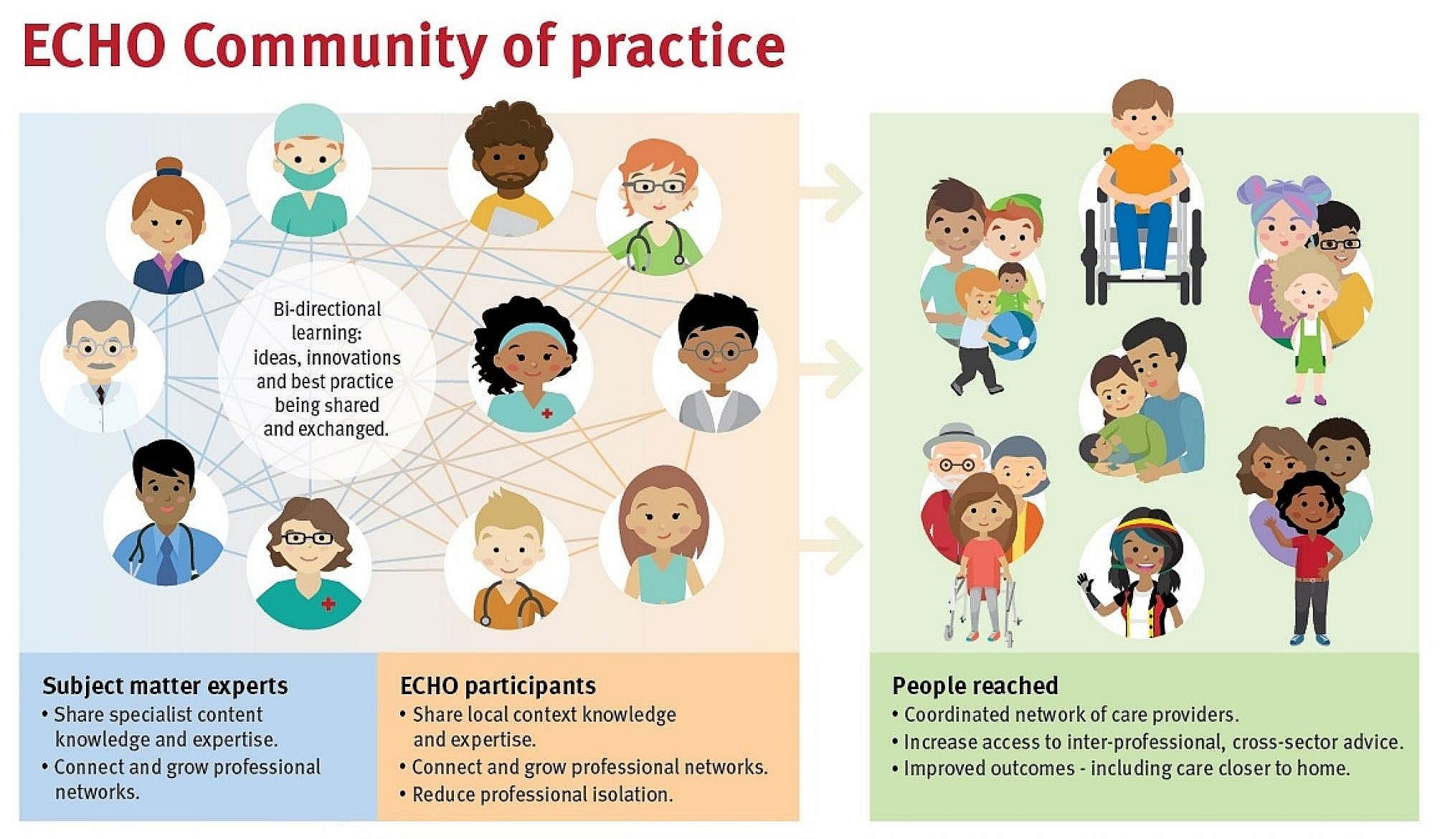
Example of how an ECHO^®^ Network functions in practice. Copyright: Children’s Health Queensland Hospital and Health Service, used with permission

**Fig. 2:**
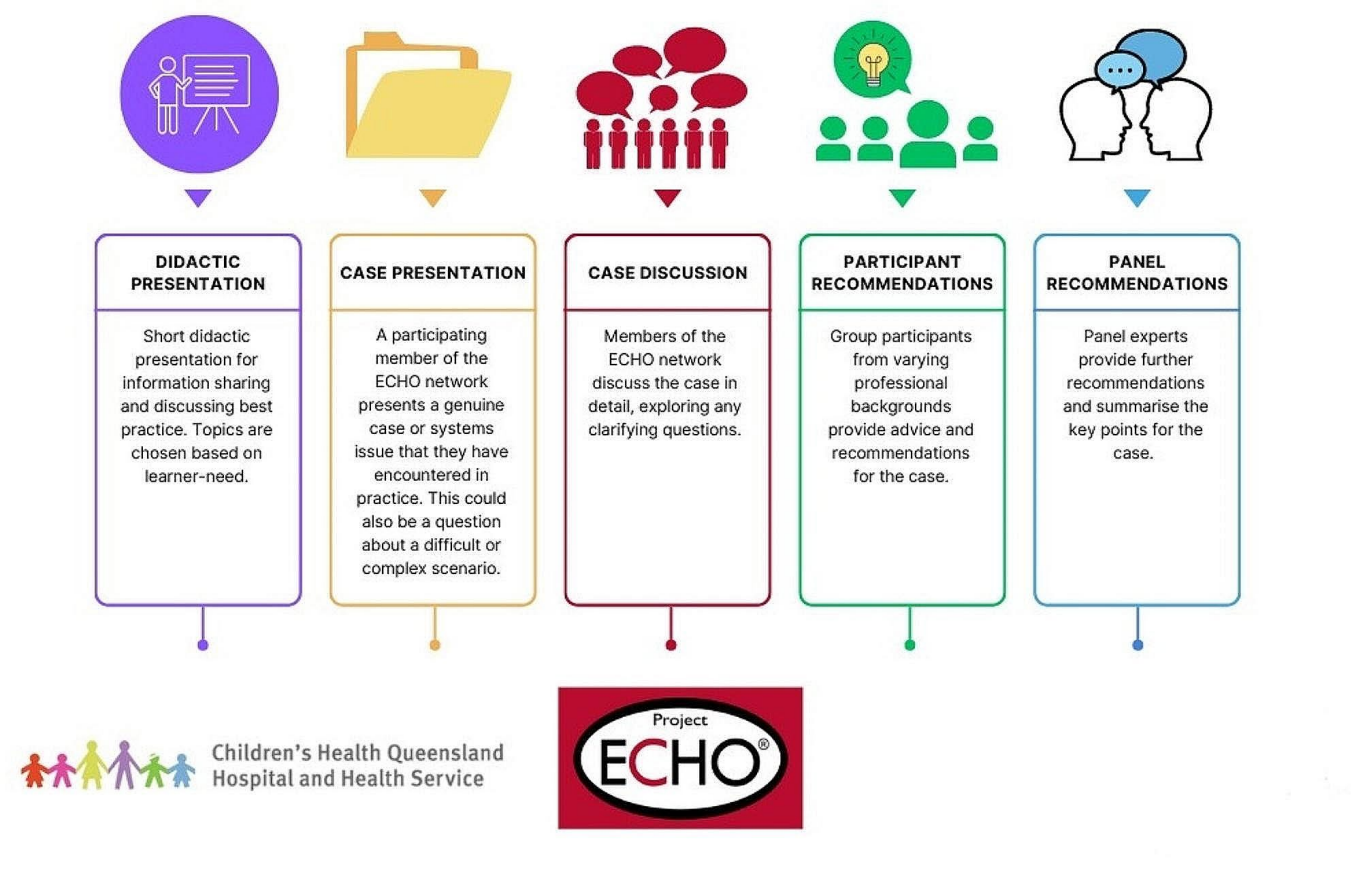
High-level structural overview of how an ECHO^®^ Network session is conducted. Copyright: Children’s Health Queensland Hospital and Health Service, used with permission

Organizations from any sector or geographic location can acquire a free license from the ECHO Institute™ to use the ECHO model™ as a framework and toolkit to independently establish their own virtual communities of practice or ECHO^®^ Networks as they are more commonly known. Organizations use these ECHO^®^ Networks as enablers to build workforce capacity, capability, confidence, and system integration by scaling scarce subject matter expertise to enhance service provision at the point of care through these virtual communities of practice [29]. This is achieved through each ECHO^®^ Network functioning as a virtual, non-hierarchical, bi-directional knowledge sharing forum that can evolve over time to remain responsive to the network membership’s learning objectives, mentorship needs, and the ECHO^®^ hub’s organizational landscape [29]. Figure 1 below illustrates how an ECHO^®^ Network functions in practice. Routine ECHO^®^ Network sessions feature short, best-practice presentations, case-based discussions centered around real participant experiences, and offer opportunity for professionals to connect and collaborate in a free and convenient virtual format [32]. Figure 2 below illustrates a high-level structural overview of how an ECHO^®^ Network session is conducted.

Each organization that acquires a license to implement and use the ECHO model™ is referred to as an ECHO^®^ hub. Depending on the strategic and/or operational priorities and available resources of the hub organization, it may launch and operate any number of different ECHO^®^ Networks. Each ECHO^®^ Network has an overt focus area, such as Hepatitis C, Pediatric ADHD, Child Protection, Refugee/Multicultural Health, and so on, which is purposeful to attract a specific target audience of participants, known as spokes, to join the ECHO^®^ Network.

In addition to the ECHO Institute™ at the University of New Mexico licensing new organizations to become ECHO^®^ hubs, the ECHO model™ is further diffused by established ECHO^®^ hub organizations known as Superhubs [33]. Superhubs are ECHO^®^ hubs which have had a successful track record in implementing and sustaining the innovation, who have acquired an additional licensing designation from the University of New Mexico’s ECHO Institute™ to become an ECHO^®^ Superhub [29]. ECHO^®^ Superhub organizations function as regional centers of excellence to support other organizations to adopt and implement the ECHO model™ as a new innovation. ECHO^®^ Superhubs provide formal partner launch training (also known as Immersion), mentor new implementation teams to adopt and establish ECHO^®^ hubs in their own organizations, and offer ongoing monitoring and fidelity assurance support to increase rates of diffusion.

Within the healthcare sector, organizations that have implemented the ECHO model™ have demonstrated varied improvements in healthcare service delivery, workforce development and patient outcomes [34–38]. Despite a significant global diffusion of the ECHO model™ across more than 1000 organizations in 68 countries [30], there remains a gap in the literature as to the organizational and team factors that can influence successful implementation of this innovative model [39]. To address this gap, this study builds upon previous research which identified indicators of implementation success by measuring them within a global sample of ECHO^®^ hub organizations [39].

### Context and aims of study

This study seeks to contribute new evidence by examining implementation success indicators of this new innovation model to achieve two key aims:

1. To understand how new innovation implementations vary across key success indicators within a global sample, and
2. Identify and understand what enablers/barriers exist for organizational teams in implementing new innovations.

In response to the study’s two aims, the first objective of this study was to analyze what the current state of implementation success is for Project ECHO^®^ implementations globally, to determine how they compare across countries and sectoral contexts. This analysis sought to provide the first known cross-sectional description of Project ECHO^®^ implementations, synthesizing new international evidence. Further, this analysis sought to provide a description of the variations of Project ECHO^®^ implementations that were drawn from a multi-country sample to highlight variances and commonalities. The second objective of this study was to empirically examine insights that would assist organizational teams in successfully implementing Project ECHO^®^. While the study sought to examine success indictors aligned with the implementation of this innovative model, there was also an opportunity to glean quality improvement and implementation support resourcing insights that would enhance subsequent implementations of Project ECHO^®^ globally. In addressing the two aforementioned key aims, this study highlights salient aspects that exemplify implementation success for Project ECHO^®^ and seeks to strengthen the case for organizations to implement this model as a new innovation.

Due to the significance of implementation failure and the rising operating costs of organizational expenditure across sectors globally, it is essential for organizational decision-makers to understand how they can embed and leverage innovations such as Project ECHO^®^ to enhance the way their services operate and integrate across the system [1, 40]. This study makes an empirical contribution to the literature by explaining the process by which organizational teams can measure and report their implementation success using a universal framework of indicators with real-world examples from the field. It also addresses a gap in the literature to better understand how innovations such as Project ECHO^®^ can be implemented and embedded within organizations successfully. Findings from this study are also anticipated to provide practical strategies and insights to support executive decision-makers and implementation teams to enhance the successes of future implementations of the ECHO model™.

## Methods

### Study design

This empirical study used a cross-sectional research design [41–46] to collect and report implementation and demographic data from a global sample of organizations which had implemented Project ECHO^®^. This study design was selected in order to collect and analyze a large volume of data from the target population at a single point in time and subsequently facilitate describing key findings. The study design employed an online survey format to collect data on 54 indicators of implementation success which consisted of a total of 78 questions. These indicators (see Additional Information File I) were derived from previous research led by the authors of this study through an e-Delphi process whereby a panel of international Project ECHO^®^ experts participated in the nomination, refinement, and rating of implementation success indicators of Project ECHO^®^ [39]. As such, there was no previously validated data collection tool in existence to utilize, thus a custom data collection survey tool was developed by the authors. This two-part survey was administrated through the Qualtrics online platform to allow for the collection of data from a global sample in two parts (see Additional Information File II). Part A of the survey was designed to measure specific implementation success indicators that related to the overarching ECHO^®^ hub’s organizational context. This was captured as a once-off submission per each organization that participated. Subsequent to this, the Part B component separately measured outcomes of each individual ECHO^®^ Network operated by these ECHO^®^ hubs, allowing for each hub to make multiple submissions where they operated more than one ECHO^®^ Network. The cross-sectional design was thus selected in order to establish baseline evidence of the first international state of play for Project ECHO^®^ implementations by harnessing the significant volume of data to be collected in this study.

### Participants and sampling

At the time of this study, global adoption of Project ECHO^®^ had extended to 68 countries and consisted of 1000 + hub organizations which had implemented the ECHO model™. These organizations served as the potential sample population for this study. A convenience sampling approach was selected whereby implementation teams from all of these organizations were invited to opt-in to participate in this study via an advertisement placed in the ECHO Institute™ fortnightly e-newsletter. The invitation to participate in the research study was included in the ECHO Institute™ newsletter on four occasions during the three-month data collection period (June-September 2022). Fortnightly email reminders were sent to participants who had initiated a response to the invitation to complete their organization’s survey submission.

The ECHO Institute™ newsletter is a long-established, well-governed, and familiar communication channel used by other licensed Project ECHO^®^ hubs globally to communicate about their local implementation and research activities. The newsletter medium was seen as a strategic and practical communication pathway by which to provide access to and increase visibility of the research study while removing any perceivable risk that the survey invitation might inadvertently be considered spam by the prospective research participants.

The newsletter was distributed to a global readership of Project ECHO^®^ practitioners employed within these organizations. Subsequent to the initial convenience sampling approach, a snowball sampling method was also employed by encouraging potential implementation teams to share the invitation with colleagues in other Project ECHO^®^ hub organizations whom they believed would also be interested in participating. The newsletter invitation included hyperlinks to the research study information, survey completion guide, and consent forms which were embedded on the landing page of the Qualtrics survey portal. The survey completion guide was a fillable PDF document which replicated the Qualtrics online portal, to allow participating implementation teams to collate the data for submission offline using the template and submit online via Qualtrics when all the requisite data had been collected.

No minimum sample size was required for this study, however, sufficient participation to achieve variance (by geography, sector, organization type, etc.) was a key focus of the research team. Participant variance would be monitored in Qualtrics throughout the data collection phase to identify if additional promotional efforts were required, and to determine when study enrolment saturation occurred. Following their participation, ECHO^®^ hub teams would be provided with individualized reports of their organization’s local ‘state of play’, contrasted with aggregated de-identified global results to enable local benchmarking and subsequent performance improvements. These individual reports would include comparison averages and inter-quartile ranges from all respondents and be released after the study’s completion.

### Measurement and data collection

The two-part custom survey consisted of 78 questions adapted from a framework of 54 indicators to measure implementation success for Project ECHO^®^ [39]. These indictors sought to measure implementation success metrics across four key domains, outlined with definitions below:

1. Participant Engagement– 14 indicators which measured the number, interactivity and participation experience of individuals who join ECHO^®^ Networks from a variety of geographic/sectoral locations to connect and learn with panel teams centrally coordinated by the hub.
2. ECHO^®^ Hub/ECHO^®^ Network design and operation– 23 indicators which measured the design and operation of an organization’s ECHO^®^ hub, and/or individual ECHO^®^ Networks.
3. ECHO^®^ Hub team engagement– 5 indicators which measured the number, interactivity, and participation experience of organizational team members who facilitate and manage ECHO^®^ hub functions, and
4. Local Impact– 12 indicators which measured the increase or improvement in workforce development, capacity, system integration and efficiency [39].

**Fig. 3:**
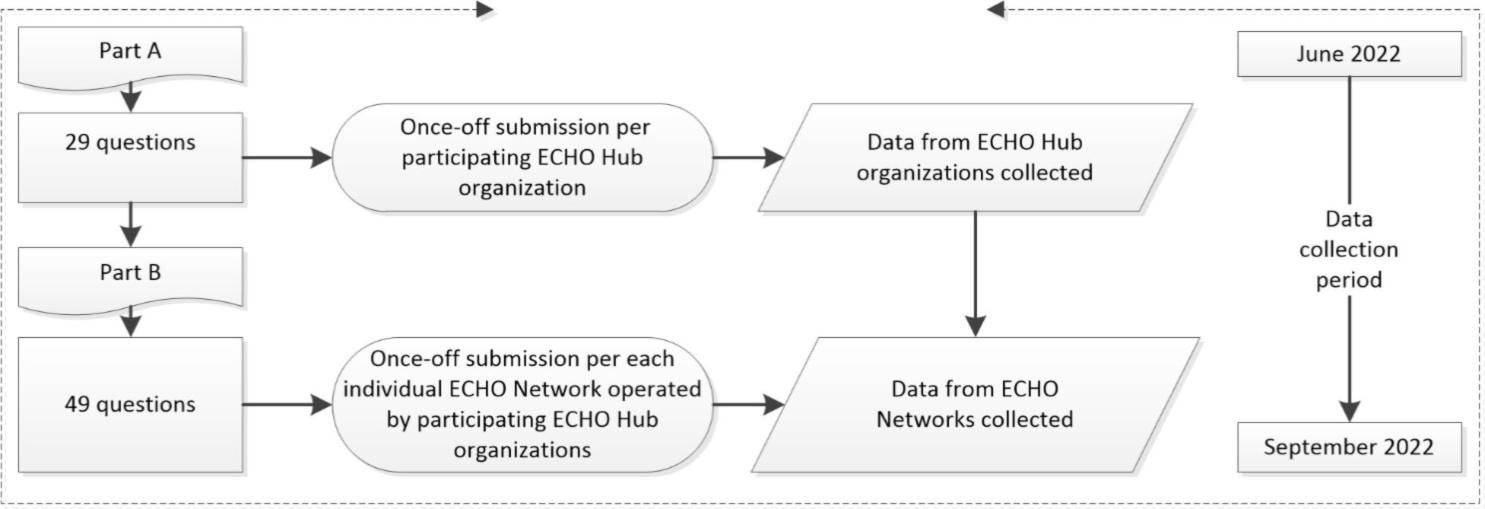
Survey design and data collection process

Due to the implementation success indicators for Project ECHO^®^ being designed to measure the innovation’s organizational and individual ECHO^®^ Network specific outcomes, the survey was administered in two parts, with Part A and B incorporating indicators across each of the above domains as relevant, as illustrated in Fig. 3.

Part A collected organizational and demographic data for each Project ECHO^®^ hub that participated. This data was only required as a once-off, single point-in-time data submission per Project ECHO^®^ hub organization. There were 29 questions in Part A, which elicited organizational-specific demographics and indicators for measurement. The 49-question Part B component facilitated the collection of data from multiple individual ECHO^®^ Networks for each specific virtual community of practice that was operated by the respective Project ECHO^®^ hub organization. Responses to both Part A and Part B surveys consisted of free-text, multiple choice and 10-point Likert scale response fields. Having this two-part capability within the survey ensured a convenient and straightforward way for study participants to submit data where hub organizations operated more than one ECHO^®^ Network.

Data for this study was collected between June and September 2022. Data was collected in each participating Project ECHO^®^ hub organization by a nominated lead respondent using the fillable survey completion guide, then submitted via the online survey. The data collection phase lasted for three months to maximize the potential research participants to opt-in to the study, and for them to gather as much implementation data as possible. This acknowledged that the indicators used in this study extended beyond basic metrics captured by the current iECHO client relationship management (CRM) system used by all Project ECHO^®^ hub organizations around the world. Each part of the surveys took organizational leads approximately 20–30 min to complete by inputting the data collated in the survey completion guide.

### Data analysis – statistical methods

Quantitative data was extracted from Qualtrics and cleaned using Microsoft Excel. Descriptive statistical analyses were undertaken using the SPSS software package. The data were analyzed to identify characteristics and variations using descriptive statistics (i.e., averages, inter-quartile ranges, trendlines) to profile the various implementation characteristics that emerged from the data and to compare individual Project ECHO^®^ hub or network results per indicator. Where any survey question elicited participants to provide an ‘other– please specify’ response, which was clarified with optional text, the text component was analyzed and reported descriptively.

#### Reporting back to study participants

Following the completion of the data analysis phase, individualized reports were produced and released to each of the participating Project ECHO^®^ hub implementation teams, with the offer for the Principal Investigator to meet via Zoom to discuss the results and provide any clarification regarding the aggregate results from the sample.

#### Ethical Statement

Ethical approval to undertake this research was provided by both The University of Queensland and Children’s Health Queensland Hospital and Health Service’s Human Research Ethics Committees (HREC/22/QCHQ/86,967, 2022/HE001044, and SSA/2022/QCHQ/86,967).

## Results

### Study population

Out of the total 1000+ Project ECHO^®^ hub organizations across 68 countries, this study attracted a total of 13 hubs to participate from five countries. This was accepted as sufficient participant variance, without requiring additional promotional efforts. The highest participation rate was from implementation teams employed within seven organizations across Australia (*n* = 7, 54%). These organizations were licensed to and had implemented Project ECHO^®^ as a new innovation between 2016 and 2022. From the 13 Project ECHO^®^ hub organizations data was also gleaned from 51 individual ECHO^®^ Networks which were operated by these hub organizations.

**Table 1:** ECHO^®^ Networks– total launched versus sustained (Part A data)

Number of ECHO^®^ Networks operated by Hubs		
Hub	Total Networks launched	Active Networks (sustained)
Hub 1	2	1
Hub 2	1	1
Hub 3	1	1
Hub 4	14	14
Hub 5	12	5
Hub 6	5	2
Hub 7	21	12
Hub 8	1	1
Hub 9	1	1
Hub 10	8	3
Hub 11	2	2
Hub 12	8	7
Hub 13	1	1
Total	77 Total ECHO^®^ Networks Mean: 6, Min: 1, Max: 21.	51 Active ECHO^®^ Networks at time of data collection Mean: 4, Min: 1, Max: 14.

The results of these survey data from Part A (overall ECHO^®^ hub organizations) and Part B (individual ECHO^®^ Networks) elicited from the participating hubs are discussed in the next two sub-sections to align with the study aims. The following results provide a cross-sectional description of the participating organizations which have implemented Project ECHO^®^ with summary data presented in Tables 1 and 2.

**Table 2:** ECHO^®^ Hub organization overview (Part A data)

	Hub Count	Percentage
**Country of origin**		
Australia	7	54%
United States	2	15%
Canada	2	15%
India	1	8%
Sudan	1	8%
Total	13	100%
**Hub Sector**		
University (Research/Academic, non-service delivery)	2	15%
Healthcare (Tertiary/Quaternary)	4	31%
Healthcare (Secondary)	1	8%
Healthcare (Primary)	4	31%
Healthcare (Public Health)	2	15%
Total	13	100%
**ECHO** ^**®**^ ** Replication Phase**		
Launch	1	8%
Growth/Continuous Improvement	12	92%
Total	13	100%
**Immersion training– year completed**		
2016	1	8%
2017	3	24%
2018	2	15%
2019	1	8%
2020	2	15%
2021	2	15%
2022	2	15%
Total	13	100%
**Number of Superhub engagements within first 12 months post-Immersion training**		
0–10 engagements	7	54%
11–20 engagements	3	23%
21–30 engagements	2	15%
31 + engagements	1	8%
Mean: 18.8, Min: 0, Max: 100		100%
**ECHO** ^**®**^ ** Hub– total FTE staffing**		
0–3	11	84%
8	1	8%
13	1	8%
Mean: 2.9, Min: 1, Max: 13		100%
**Number of disciplines involved in ECHO** ^**®**^ ** Hub activities, per organization**		
0–5	8	61%
5–10	4	31%
10+	1	8%
Mean: 6.08, Min: 1, Max: 23		100%
Pilot Funding Sources*		
None	1	6%
Organizational Funding– temporary	4	24%
Organizational Funding– permanent	3	18%
Competitive Grant	3	18%
Commissioning / Contracted Funding	6	35%
Discretionary Operational Funding	1	6%
Mean: 1.4, Min: 0, Max: 4		100%
Ongoing Funding Sources*		
None	2	7%
Organizational Funding– temporary	5	19%
Organizational Funding– permanent	5	19%
Competitive Grant	5	19%
Commissioning / Contracted Funding	6	22%
Philanthropic Grant	3	11%
Discretionary Operational Funding	1	4%
Mean: 2, Min: 0, Max: 6		100%
Governance Processes*		
None	2	6%
Decision-Making Framework	5	14%
HR/Professional Line Management Procedures	8	23%
Organizational Leadership Role Oversight	10	29%
Advisory Group / Governance Forum	2	6%
Financial Management / Delegations Framework	8	23%
Mean: 2.7, Min: 0, Max: 4		100%
Executive/Leadership support*		
Organizational framework/reports	11	28%
Legal Documentation	5	13%
Project Documentation	9	23%
Briefing Notes or other official correspondence	13	33%
Email Correspondence	1	3%
Mean: 3, Min: 1, Max: 5		100%
Marketing Processes*		
Marketing Strategy / Plan	6	13%
Information Flyer	8	17%
Website	13	27%
Social Media posts (LinkedIn, Facebook, Twitter, other)	10	21%
Formal Media Release	2	4%
Email Newsletters / Broadcasts	6	13%
Stakeholder Meetings	1	2%
Simulation Events	1	2%
Word of Mouth	1	2%
Mean: 3.7, Min: 2, Max: 6		100%
Data Collection Processes*		
Spoke participant registration lists	12	21%
iECHO CRM (licensed CRM)	12	21%
CRM– other than iECHO (licensed CRM)	4	7%
Case Presentation Templates	6	11%
Consent Protocols	10	18%
Research Ethics Approvals	7	12%
Event Summary Reports	1	2%
Online Survey Platforms	1	2%
Online CME tracking platform	1	2%
Online Self-Assessment Tools	1	2%
Microsoft Excel	2	4%
Mean: 4.4, Min: 2, Max: 7		100%

*NB: these results illustrate where individual ECHO^®^ hub organizations evidenced multiple sources

While the study was open to Project ECHO^®^ hub organizations globally and from any sector, there was strong representation of hub organizations from the healthcare and university sectors, particularly in Australia. This result has reinforced the relevance of the ECHO model™ as an accepted innovation to build workforce capacity, capability, confidence, and system integration within the broader healthcare sector.

### Financial landscape

Funding sources for implementations were almost unanimously temporary in nature, with commissioning, contracted or grant funding being the most common investment streams for Project ECHO^®^ hub teams to pilot the innovation within their organizations. Despite this financial landscape, all Project ECHO^®^ hubs (100%) were able to evidence transparent forms of financial governance, either in the form of financial reports/statements documenting the administration of Project ECHO^®^ implementation funding, or ongoing organizational cost center reports.

### ECHO^®^ hub implementation activities

Prospective organizations are required to complete a once-off compulsory hub launch readiness training program (also known as Immersion), prior to becoming licensed to implement and use the ECHO model™ [39]. Immersion training is delivered by well-established ECHO^®^ Superhub organizations, which provide ongoing partner liaison, mentorship, and implementation support to new implementation teams adopting the ECHO model™ within their organizations. Across the sample, implementation teams from all organizations (*n* = 13, 100%) reported they had completed the Project ECHO^®^ Immersion training to support their local implementation efforts, with each hub organization having on average five staff trained. With a minimum of 1 staff member per organization mandated to complete Immersion training as part of the Project ECHO^®^ licensing requirements, three organizations (23.1%) invested in sending 8, 10 and 13 staff respectively.

Implementation of the ECHO model™ typically occurs across three key phases, (1) pre-launch– the period immediately following the completion of Immersion training up until the date the hub organization launches their first pilot ECHO^®^ Network session; (2) launch– the duration of the ECHO^®^ hub organization’s initial pilot ECHO^®^ Network; and (3) growth/continuous improvement– the ongoing period thereafter. In this sample, all but one (*n* = 12, 92%) organization was in the growth/continuous improvement phase of their Project ECHO^®^ implementation, meaning they had implemented and launched at least one ECHO^®^ Network. These organizations all reported progressing along the implementation continuum from pre-launch to the final phase of growth/continuous improvement within 12–18 months from completing Immersion training.

There appeared to be a consistent relationship between Project ECHO^®^ hub organizations which engaged more with their Superhub training provider, launching higher numbers of ECHO^®^ Networks. For implementation teams which engaged with their Superhub more than 20 times within the first 12 months of completing Immersion training (*n* = 5/13, 38.5%), the average number of total ECHO^®^ Networks launched was 13, compared to the total sample of 5.9 ECHO^®^ Networks. Implementation teams who engaged more with Superhubs (*n* = 5/13, 38.5%) were also observed to sustain a higher number of currently active ECHO^®^ Networks, with an average of 6.4 active ECHO^®^ Networks, compared with 3.9 ECHO^®^ Networks across the total sample.

The data collected also highlighted those organizations (*n* = 10/13, 77%) which undertook more time (greater than five months) for pre-launch planning activities, following the completion of initial Immersion training, were observed to launch on average three times as many ECHO^®^ Networks in total compared to their peers (*n* = 60/77, 78%). Furthermore, this group also demonstrated a greater ability to sustain more active ECHO^®^ Networks over the longer term than those hubs which only launched a single ECHO^®^ Network shortly after completing Immersion training (within less than five months) (*n* = 39/51, 77%). Table 1 illustrates the spread of total launched (*n* = 77) versus sustained (*n* = 51) ECHO^®^ Networks for each participating organization.

### Staffing

In addition to the dedicated Project ECHO^®^ hub operational FTE staff, the number of unique individuals that fulfilled Project ECHO^®^ activities within the wider organization was much larger. These individuals typically fulfilled ECHO^®^ Network panel expert roles in a partial FTE capacity or on an hourly basis. Similarly, there was variation across the sample with most hubs having modest numbers of individuals involved (< 18 individuals, *n* = 10, 76.9%), while three hubs (23.1%) reported numbers of 30, 40 and 73 respectively. In addition to professional diversity, Project ECHO^®^ hub teams reported a strong understanding, on average 8.8/10 (*n* = 13) (0 = Very Low, 10 = Very High), of the theoretical and practical elements of implementing and using the ECHO model™ within their organization to achieve benefits in the local context.

### Governance and fidelity assurance

Evidence of executive/leadership support of Project ECHO^®^ implementations varied across the sample. There was variation in governance processes across hubs, with five sites (38.5%) reporting having minimal or no governance oversight within their organizations. The remainder reported having well-established governance and oversight processes for the implementation and operation of their Project ECHO^®^ hubs. Eight hubs (61.5%) reported having a mix of three or more governance and oversight processes in place to support their implementation.

It was also quite common for Project ECHO^®^ hubs to utilize a suite of documented operational processes to support their implementation and fidelity assurance processes. Organizations reported the use of five processes on average, including work instructions, procedures, quality assurance scorecards, templates, and consent forms. There was no correlation for hubs that sustained the operation of multiple ECHO^®^ Networks having higher numbers of documented operational processes in place.

### Marketing and data collection activities

On average, each hub reported utilizing a mix of marketing processes to promote their Project ECHO^®^ operations. Eleven hubs (85%) also reported evidence of internal stakeholders from within Project ECHO^®^ hub organizations advocating to prospective spoke participants, recommending that they join the ECHO^®^ Networks offered by the hub organization. However, one indicator also identified that the majority of ECHO^®^ Networks within the sample (37/51, 72.6%) did not collect evidence of peer-to-peer testimonials where spoke participants promoted their positive experiences or encouraged other colleagues to join ECHO^®^ Networks. In terms of data collection processes that focused on collecting implementation outcomes, participating hubs reported having multiple processes in place.

The remaining results that follow in this section pertain more specifically to the data of 51 ECHO^®^ Networks that were reported by the 13 ECHO^®^ hub organizations which participated in this study.

### Age and composition of ECHO^®^ networks

The average age of an ECHO^®^ Network across the sample was 2.16 years, with some operating for more than six and a half years, and some less than six months. Over half of the sample ECHO^®^ Networks were either currently operating (18/51, 35%), or temporarily paused due to routine hiatuses (19/51, 37%), with 28% (14/51) of the sample representing ECHO^®^ Networks that had concluded. The regular frequency for which ECHO^®^ Networks held sessions varied across the sample. While weekly (23/51, 45.1%), fortnightly (14/51, 27.5%), and monthly (9/51, 17.7%) were the most common frequencies, other frequency variations existed including twice-weekly (*n* = 1, 2%), third-weekly (every three weeks) (*n* = 1, 2%), six-weekly (every six weeks) (*n* = 2, 4%), and quarterly (*n* = 1, 2%). The operating cycles for ECHO^®^ Networks within the sample revealed that the majority employed a cohort approach (29/51, 56.9%), or a continuous/ongoing approach (20/51, 39.2%). The remaining three ECHO^®^ Networks employed a hybrid mix of cohort, continuous and drop-in approaches for managing spoke participant membership.

Attendance rates at sample ECHO^®^ Networks averaged at 216 individual spoke participants since launching and an average of 111 within the last 12 months, with approximately 34 spoke participants attending each session. Across the sample, an average of 28% of individual spoke participants attended the majority (over 80%) of ECHO^®^ Network sessions. Older ECHO^®^ Networks which had been running for longer periods of time (*n* = 4, 7.8%) had rates ranging between 490 and 822 individual spoke participants, while newer launches (*n* = 47, 92.2%) were in the range of between 15 and 90.

Across the sample, each Project ECHO^®^ hub attracted spoke participants to their ECHO^®^ Networks from a minimum of three different sectors, on average 6.6 sectors per hub, with two reaching 14 and 17 sectors respectively. Of the twenty sectors that were reported, Healthcare (40/51, 78.4%), Professional Bodies (Representative/Training Provider) (5/51, 9.8%), and Research Institutes (including Clinical Trials) (5/51, 9.8%) were the most common organizational sectors targeted for participation by Project ECHO^®^ hubs to attract to their ECHO^®^ Networks.

The diversity of spoke participants’ professional disciplines was on average 8.1 disciplines per ECHO^®^ Network. No data was reported on gender, race/ethnicity, or geographical diversity due to the limited capability of the iECHO CRM system. However, study respondents self-reported a mix of geographic distribution of spoke participants ranging from metro/urban, regional, rural, remote, and international in each ECHO^®^ Network.

The total number of spoke participants who presented cases for advice and support from the panelists and other spoke participants per ECHO^®^ Network since launch varied considerably across the sample with an average of 17.9 individuals per ECHO^®^ Network. On average, 15.22% of cases were represented across the sample of ECHO^®^ Networks (presented for discussion, advice, support more than once), with all but one (*n* = 50, 98%) of the sample reporting representation rates in the 0–20% vicinity.

### ECHO^®^ network participant experience and impacts

Table 3 presents the results from a number of single session polls which were completed by ECHO^®^ Network participants to report on particular experience and satisfaction indicators from Part B of the survey. These results establish a baseline score for which participating hub organizations could measure changes for quality improvement into the future. Across these 22 indicators, the sample results illustrate consistently high to very high rates of participant experience and satisfaction with the various aspects of their participation in ECHO^®^ Networks. Depending on the purpose and aims, which varied per each individual ECHO^®^ Network, not all of these indicators may have been universally relevant. For example, the indicator to measure changes in professional isolation for participants in an ECHO^®^ Network designed for metropolitan professionals which already have routine access to interprofessional colleagues may be less relevant, but not necessarily obsolete. However, these indicators reinforce opportunity to capture nuances that exist and showcase where variations or areas for improvement have been found to allow for consistent and reliable comparisons and contrasts to occur.

**Table 3:** ECHO^®^ Network participant experience and satisfaction indicators– *n* = 51 (Part B data)

Indicator	Average Result (10-point Likert scale)	Scale parameters
Participant experience	8.51/10	(0 = Not Very Enjoyable, 10 = Very Enjoyable)
Participant safety and comfort– in presenting cases	8.11/10	(0 = Not Very Safe/Comfortable, 10 = Very Safe/Comfortable)
Participant satisfaction with didactic content	8.83/10	(0 = Not Very Satisfied At All, 10 = Very Satisfied)
Participant satisfaction with panel expertise and hub support	8.87/10	(0 = Not Very Satisfied At All, 10 = Very Satisfied)
Participant satisfaction with case discussions (overall learning, advice, support gained from case presentation, discussion and recommendations developed within the ECHO^®^ Network sessions)	8.84/10	(0 = Not Very Satisfied At All, 10 = Very Satisfied)
Participant satisfaction with in-session dialogue– asking questions	8.69/10	(0 = Not Very Satisfied At All, 10 = Very Satisfied)
Participant satisfaction with in-session dialogue– making recommendations	8.65/10	(0 = Not Very Satisfied At All, 10 = Very Satisfied)
Participant safety and comfort– attending sessions– feeling safe	8.83/10	(0 = Not Feeling Very Safe At All, 10 = Feeling Very Safe)
Participant safety and comfort– attending sessions– feeling supported	8.75/10	(0 = Not Feeling Very Supported At All, 10 = Feeling Very Supported)
Participant safety and comfort– attending sessions– feeling welcomed	8.75/10	(0 = Not Feeling Very Welcomed At All, 10 = Feeling Very Welcomed)
Participant satisfaction– balance in dialogue	8.7/10	(0 = Not Very Satisfied At All, 10 = Very Satisfied)
Participant satisfaction– non-hierarchical forum	9.06/10	(0 = Not Very Satisfied At All, 10 = Very Satisfied)
Participant confidence	8.12/10	(0 = Very Low, 10 = Very High)
Participant competence	8.22/10	(0 = Very Low, 10 = Very High)
Participant knowledge/skills	8.44/10	(0 = Very Low, 10 = Very High)
Participant capacity	7.31/10	(0 = Very Low, 10 = Very High)
Participants becoming local experts	7.48/10	(0 = Very Low, 10 = Very High)
Participant self-efficacy	8.09/10	(0 = Very Low, 10 = Very High)
Participant professional isolation (positively impacted by participation in ECHO^®^ Networks)	7.7/10	(0 = Very Low, 10 = Very High)
Participant joy of work	7.78/10	(0 = Very Low, 10 = Very High)
Participant knowledge sharing relationships	7.68/10	(0 = Very Low, 10 = Very High)
Participant satisfaction– service improvements observed as impacted by ECHO^®^ participation	6.93/10	(0 = Very Low, 10 = Very High)

### ECHO^®^ network panelist experience and impacts

Table 4 presents the results from a number of single session polls which were completed by ECHO^®^ Network panelists to report on specific experience and satisfaction indicators from Part B of the survey. These results also establish a baseline score for which participating hub organizations could measure changes for quality improvement into the future. These results suggest a strong degree of positive experience and satisfaction for internal organizational adoption and acceptability of Project ECHO^®^ as a new innovation.

**Table 4:** ECHO^®^ network panelists’ experience and satisfaction indicators– *n* = 51 (Part B data)

Indicator	Average Result (10-point Likert scale)	Scale parameters
Panelist experience and satisfaction (enjoyment, high value, time efficient)	8.96/10	(0 = Not Very Satisfied At All, 10 = Very Satisfied)
Panelist satisfaction with Network Facilitation	8.7/10	(0 = Not Very Satisfied At All, 10 = Very Satisfied)
Panelist satisfaction with Panel Cohesion	8.84/10	(0 = Not Very Satisfied At All, 10 = Very Satisfied)
Panelist satisfaction with Session Satisfaction	8.8/10	(0 = Not Very Satisfied At All, 10 = Very Satisfied)
Panelist recruitment and retention– Champion	8.5/10	(0 = Not Very Satisfied At All, 10 = Very Satisfied)
Panelist recruitment and retention– Panel membership	8.5/10	(0 = Not Very Satisfied At All, 10 = Very Satisfied)
Panelist satisfaction with case discussions	8.61/10	(0 = Not Very Satisfied At All, 10 = Very Satisfied)

The following results provide a description of the enablers/barriers that existed from the 13 participating organizations which have implemented Project ECHO^®^. These results were derived from the Part B component of this study’s survey and contribute to providing a description of what is and is not working well from a multi-country sample of 51 ECHO^®^ Networks.

### Project ECHO^®^ hub team diversity and sector focus

Similarly to the diversity amongst spoke participant sectors in the results above, ECHO^®^ hub organizations also had professional diversity. Each organization reported an average of six disciplines involved in ECHO^®^ hub operations, with one organization reporting 23 different disciplines within the hub team. Administration (11/51, 21.6%), Medical– General (9/51, 17.7%), Nursing– General (5/51, 9.8%), and Psychology (5/51, 9.8%) were the most common professional disciplines involved in Project ECHO^®^ hub operations across the sample. Despite this diversity, a significant barrier that existed across the sample was access to research and evaluation resourcing. In this study, only one hub organization (1/13, 7.7%) reported having access to research, evaluation, or librarian disciplines within their hub team.

### ECHO^®^ network pre-launch planning

Pre-launch planning processes were common but varied across the sample of ECHO^®^ Networks, with an average of 5.3 processes per ECHO^®^ network occurring. These included sending additional staff to complete Immersion training (*n* = 23, 45%), local internal ECHO^®^ onboarding training for ECHO^®^ Network coordinators (*n* = 30, 59%) and panelists (*n* = 48, 94%), running mock ECHO^®^ Network sessions (*n* = 44, 86%), developing implementation (*n* = 37, 73%) and evaluation plans (*n* = 43, 84%), and undertaking learner needs assessments (*n* = 45, 88%) to inform curricula development for ECHO^®^ Networks. The most common pre-launch planning activities were panelist onboarding (*n* = 48/51, 94%), learner needs assessments (*n* = 45/51, 88%) and mock ECHO^®^ sessions (*n* = 44/51, 86%). The value in undertaking these pre-launch planning activities is covered in depth in the Immersion training curriculum as a key tenet of enhancing fidelity assurance and longer-term sustainability of new ECHO^®^ Networks beyond pilot phase. However, these results highlighted that while some processes were implemented for each ECHO^®^ Network, it was not consistent across the sample. Only one third (*n* = 17/51, 33%) of all ECHO^®^ Networks utilized all seven processes despite them being recommended as core in the Immersion training curriculum delivered by ECHO^®^ Superhubs [29]. This variation in implementation planning may present a barrier for ECHO^®^ hub implementations to be benchmarked effectively and may erode their fidelity assurance and sustainability beyond pilot phase.

On average, ECHO^®^ Networks utilized more than one (1.5) fidelity assurance tools, ranging from the Anatomy of an ECHO^®^ tool, ECHO^®^ session scorecard, facilitator scorecard, and Superhub observation and mentorship to ensure the design and delivery of sessions was aligned to the ECHO model™. The most strongly utilized tool was the Anatomy of an ECHO^®^ tool, across 47/51 (92%) ECHO^®^ Networks, followed closely by the ECHO^®^ session scorecard in 25/51 (49.02%) ECHO^®^ Networks. Interestingly, 2 out of 51 ECHO^®^ Networks (3.9%) did not utilize any fidelity assurance tools.

The co-design of each ECHO^®^ Network was, on average, in response to or in alignment with 2.87 priorities or metrics ranging from population need, service-specific priorities, workforce priorities, funding opportunities, quality indicators, organizational strategy, and government priorities. Organizational strategy (24/51, 47.1%) and government priorities (22/51, 43.1%) were the most common priorities across the sample. These co-design results indicate that implementation teams focused on ensuring their ECHO^®^ Networks aligned to the strategic priorities of their organizations and governments, consistent with the available funding streams for pilot investment. However, alignment to quality indicators (*n* = 11/51), population health needs (*n* = 12/51) and workforce priorities (*n* = 17/52) featured less prominently in the results.

In addition to the stakeholder engagement in co-design processes and strategic alignment, the desired curricula topics elicited from the target stakeholders during each ECHO^®^ Network’s learning needs assessment resulted in being incorporated into the final didactic curriculum on average in 94.8% of ECHO^®^ Networks. The co-design indicators of the 51 ECHO^®^ Networks reported the following averages for targeted stakeholder input from prospective spoke participants– 45.62, consumers– 3.75, system managers– 3.33, subject matter experts/prospective panelists– 4.06, others– 11.2.

### ECHO^®^ network launch and expansion outcomes

The average number of ECHO^®^ Networks across the sample that included a case presentation in each session was 71.8%. Almost a quarter of ECHO^®^ Networks reported 100% achievement (*n* = 12/51, 23.5%), while 10 ECHO^®^ Networks (19.6%) reported case presentation rates occurring in 50% or less of sessions. Rates of spoke participants inviting colleagues to co-present case presentations during ECHO^®^ Network sessions occurred on average in 12.21% of all sessions across the ECHO^®^ Network sample. Only 3 out of 51 (5.9%) ECHO^®^ Networks manually collected and analyzed interactivity data using locally derived tools, in the absence of any data collection/analysis capabilities in the iECHO client relationship management (CRM) solution used by all Project ECHO^®^ hub organizations. Across the total sample, 48/51 (94.1%) ECHO^®^ Networks were unable to measure changes in in-session interactivity amongst spoke participants and panelists.

In the absence of a universal tool to collect and measure interactivity data, ECHO^®^ Networks were asked to self-report any observed change in spoke participant contribution to discussions since each ECHO^®^ Network launched. Across the sample, spoke participant contributions to discussions were reported to have increased by 65% since the ECHO^®^ Network’s launch on average, with the lowest reported improvement being 25%. On average, these changes were observed to occur over a 12-month period, with some improvements reported to have been observed in as little as five months.

As a result of participating in ECHO^®^ Networks, spoke participant changes in practice averaged at 4.75 changes per participant, with 83.4% of participants reporting at least one change in practice. The most common changes reported were method/approach/procedures to undertaking assessments (53, 21.9%), information collection (44, 18.2%), and techniques for working with patients/clients/consumers (42, 17.4%). These results suggest participation in ECHO^®^ Networks yielded a number of changes in practice for individual spoke participants. However, more widespread organizational or sectoral changes were yet to be realized as evidenced in other Project ECHO^®^ literature [34, 35, 47], which may be attributable to the average length of operation for each ECHO^®^ Network (2.16 years) across the sample.

### Marketing and data collection activities

On average, each individual ECHO^®^ Network utilized a mix of 3.47 different communication and data collection processes to ensure consistent and reliable engagement with targeted stakeholders and reporting. These processes included direct communication with individuals, localized communication templates for consistent messaging, routine iECHO/other CRM use, mailing lists, and formalized strategies, plans or procedures. Email mailing lists (50/51, 98%) and routine iECHO/other CRM (46/51, 90.2%) use were the most utilized processes across the sample.

### Key findings summary

1. Despite mostly temporary organizational investment, there was strong rates of adoption and sustainability amongst organizations within the healthcare sector.
2. Organizations in the sample consistently progressed through the implementation process within 12–18 months from completing Immersion, with those taking greater than five months for pre-launch planning launching on average three times more ECHO^®^ Networks in total. This suggests that implementing Project ECHO^®^ offered a speed to value which is noteworthy, given the general prevalence for new innovations to fail.
3. Despite a positive value perception amongst executive decision-makers in front-loading larger implementation teams to complete Immersion training, there was poor application of fidelity assurance tools recommended to support implementation success in the organizational setting.
4. Hub organizations which experimented and launched multiple networks demonstrated greater ability to sustain more total active ECHO^®^ Networks compared to those which only launched one pilot network shortly after completing Immersion training.
5. ECHO^®^ Networks were consistently co-designed to align with organizational and government strategic priorities in order to attract pilot investment, this may have detracted from focusing more on quality improvement and population health priorities in order to secure funding streams.
6. The framework of 54 indicators measured reinforced the implementation of the ECHO model™ to be a relevant and acceptable innovation used by organizational teams to build workforce capacity, capability, confidence, and system integration, particularly in Australia.
7. Consistent and routine engagement between ECHO^®^ hub and Superhub mentor organizations resulted in higher rates of ECHO^®^ Network expansion and sustainability.
8. Despite peer-to-peer participant advocacy being reported as the one of the most commonly used strategies to promote ECHO^®^ Networks, the majority of hub teams reported no internal capacity or available tool to measure or analyze this indicator.
9. The diversity in internal and external stakeholders’ experience and satisfaction indicators that were measured across the sample provided a well-nuanced and positive demonstration of the acceptability and value perception.

## Discussion

The following discussion is framed around this study’s two key aims to discuss the salient insights that emerged from the data which are nested under sub-headings for clarity:

1. To understand how implementations of Project ECHO^®^ as a new innovation varied across key success indicators within a global sample, and
2. Identify and understand what enablers/barriers existed for organizational teams in implementing Project ECHO^®^ as a new innovation.

### Strong adoption amongst healthcare organizations

Of the 13 organizations that participated in this study, the majority (*n* = 11/13, 84.62%) came from the healthcare sector. This is consistent with the strong rates of global adoption and origins of the ECHO model™ first being used by healthcare providers in the United States to improve health outcomes for patients living with Hepatitis C [34]. Despite more recent trends in Project ECHO^®^’s diffusion expanding into other sectors such as education, disability, climate science and others, the healthcare sector remains a strong foothold and proof of concept for the model’s success [35, 37, 38, 47–51].

### Financial landscape

The variation in financial landscapes for each of the Project ECHO^®^ hub organizations’ ongoing funding streams reinforced the difficulty that many new innovations across sectors face in securing ongoing investment to transition into business-as-usual functions [3, 13, 20, 52]. As Project ECHO^®^ continues to be considered an innovation, disruptor, and distinct departure from the status quo, the need for implementation teams to prioritize ongoing financial sustainability beyond the pilot phase remains evident [53–55]. Consistent with the data collected from this sample, almost all implementation teams reported their pursuit of a diverse range of financial investment to fund their ongoing Project ECHO^®^ operations. The continuation of good financial governance practices that were observed in all Project ECHO^®^ hub organizations may sustain the confidence of executive decision-makers in providing ongoing investment until more recurrent investment streams can be secured.

### Immersion training

These results highlighted that executive decision-makers and leaders within hub organizations perceived a strong value in upskilling a larger number of individuals within implementation teams through the Project ECHO^®^ Immersion training to ensure successful adoption and a return on investment.

Similarly, to the pre-launch planning processes mentioned above, there appeared to be a disconnect from fidelity assurance best practices reinforced by ECHO^®^ Superhubs in the Immersion training curriculum and the results in this sample of ECHO^®^ Networks. Despite this front-end investment in human resourcing, this study’s results also highlighted evidence whereby following Immersion training, implementation teams under-utilized fidelity assurance and implementation planning resources. This disconnect between the curriculum and resource toolkit covered in Immersion training being under-utilized during implementation activities suggests enhancements may be required to facilitate stronger practical application in the field. These results highlighted that fidelity assurance processes were looked at by hub teams more as a tokenistic compliance check, rather than an integral component of implementation success and quality assurance/improvement.

As a result, it may be valuable for the Immersion curriculum and resource toolkit to be reviewed and tested periodically by ECHO^®^ Superhubs to ensure it remains contemporary and able to be contextualized by implementation teams locally.

### Implementation planning, launch, and expansion

The variation in how frequently some implementation teams in this sample accessed ECHO^®^ Superhubs for support during their implementation phase appeared to highlight where more intrapreneurially-oriented implementation teams experimented with the ECHO model™ within their organization to launch additional ECHO^®^ Networks in quick succession to their pilots [1, 56, 57]. Project ECHO^®^ intrapreneurs are those individual employees who proactively identify and seize the new innovation to harness opportunities and resources (human and capital) to implement dynamic changes, or departures from the status quo to improve outcomes and create value, which is becoming more commonplace [1, 40, 57–60]. This suggested that experimentation occurs beyond piloting merely one ECHO^®^ network or focus area, and a refinement/consolidation of efforts can take place as organizational teams build their understanding of the ECHO model™, as well as other potential factors such as funding, resourcing availability etc. As evidenced in this research, for those intrapreneurial implementation teams which accessed more support from their Superhubs, it was evident that they experienced greater success than their peers in both the expansion to and sustainability of a larger number of ECHO^®^ Networks. The key sign of ECHO^®^ hub teams taking a longer pre-launch planning phase for refinement/consolidation and engagement with Superhub ‘implementation mentors’ across the sample highlighted that implementation teams gained more experience harnessing the ECHO model™ within their organizational contexts for wider and more sustained applications [61].

These findings resonate with other literature highlighting reasons where new innovations can often fail– with many due to contextual barriers linked to a mismatch between the innovation and the organization’s cultural appetite and/or financial investment in implementation resourcing, incompatible existing systems infrastructure, inadequate planning, and a lack of sustainable business models [6, 7, 13]. Despite these barriers prevalent in the implementation science literature, all organizations reported progressing along the Project ECHO^®^ implementation continuum from pre-launch to growth/continuous improvement within 12–18 months following Immersion training. This aligns with previous research suggesting that implementation teams which engaged in experimenting with the ECHO model™ within their organizational context to explore expanded utilization of the model experienced more successful adoption and sustainability long-term [1].

Although some implementation teams progressed at a differing pace and scale, this evidence suggests that the Immersion training and ongoing mentorship available from Superhub organizations invaluably supported organizational teams to implement Project ECHO^®^ successfully, and in a timely manner. The low averages for consumers, system managers and subject matter experts/prospective panelists participating in the implementation co-design process appears to be consistent with the gap in rates of individual ECHO^®^ Network alignment to quality indicators, population health needs and workforce priorities as mentioned above. Despite these stakeholder groups and focus areas being reinforced by Superhub mentors in the Immersion training curriculum, this oversight by implementation teams may have been attributable to specific strategic organizational/government funding priorities attached to pilot funding streams. This may have also impacted the slower pace and scale of some pre-launch planning activities of new pilot ECHO^®^ Networks in this sample [29].

### Staffing

With most ECHO^®^ hub teams being a party of one or two individuals, this appeared to reinforce that these core hub staff are positioned and motivated to proactively partner with and collaborate across their organizations to interface with subject matter experts who fulfil panelist roles on ECHO^®^ Networks. The success that smaller hub teams exhibited in harnessing and scaling higher numbers of ex-officio style engagement in ECHO^®^ Network panel roles illustrated the suitability of a just-in-time delivery style business model to harness scarce resources throughout hub organizations, rather than duplicate them [20, 40, 62]. This was further reinforced by the positive outcomes reported by both spoke participant and panelist experience and satisfaction indicators that were illustrated in Tables 3 and 4.

While Project ECHO^®^ hub operations within this sample appear to largely be managed by small teams, this may have been achieved due to larger front-end investments in implementation where higher numbers of employees were found to have completed Immersion training. This was highlighted by some organizations investing in larger delegations of staff completing Immersion training prior to launching their pilots. As a result, the model appears to have been better integrated within the broader organizational context, requiring a smaller number of staff to sustain operations ongoing. This may be attributable to the smaller core hub team functioning to facilitate just-in-time access to other knowledge partners that could support ECHO^®^ Networks on an as-required basis to optimize organizational efficiencies.

### Governance

There were two organizational governance landscapes that appeared to be common amongst the sample. Firstly, some implementation teams appeared to have a degree of delegated autonomy or permission to implement and experiment widely with Project ECHO^®^ as an innovation within the organization. In other organizations across the sample, implementation teams reported to have encountered more structural or cultural barriers that required them to tailor their implementation approach. This latter scenario saw teams needing to incrementally exploit levers/opportunities that would facilitate Project ECHO^®^’s integration and subsequent adoption by the broader organization as a business-as-usual function over the longer-term. Despite this, in both of these scenarios the degree of governance oversight and practice varied by organization but did not appear to have more or less remarkable outcomes in either scenario.

### Marketing and data collection activities

In the domains of marketing and data collection processes, the localized promotional activities and limited capability of the iECHO CRM hampered implementation teams in automating their engagement activities to improve the efficiency of their work. Peer to peer advocacy was reported to be one of the most effective strategies to promote ECHO^®^ Networks, however due to hub team resourcing and skill mix, no organization in the sample had capacity to collect or analyze this data. It was also reported that the limitations in the iECHO CRM solution required all hubs to utilize a number of concurrent manual data collection and analysis processes across several other systems in absence of a single integrated and automated CRM solution.

ECHO^®^ hub teams relied on very manual and laborious administrative processes using multiple desktop solutions due to the limitations of the iECHO CRM. Similarly, for data collection activities, multiple manual processes were also relied upon due to the iECHO CRM either not having relevant data collection metrics, or an inability to aggregate and present relevant indicators that met the needs of local Project ECHO^®^ hub organizations’ internal reporting requirements.

This highlights the administrative burden which exists in absence of a fully integrated CRM platform which could centralize and automate many routine marketing, communication, and data collection processes to enhance efficiency and reliability of routine engagement with and onboarding of new spoke participants in ECHO^®^ Networks.

### Age and composition of ECHO^®^ networks

The diverse mix of ECHO^®^ Networks across the sample reinforced the dynamism of the ECHO model™ to suit a range of stakeholders’ needs, with the 51 networks ranging in age, frequency, and membership cycle. Weekly, fortnightly and monthly frequencies reinforced the ECHO Institute™’s mantra of low-dose, high frequency learning, whereby the virtual communities of practice regroup on a regular, frequent basis to build rapport and comradery amongst the learning participants and panel experts.

Consistent with the peer-to-peer advocacy appearing to provide the most targeted support for growth in newly implemented Project ECHO^®^ hubs, younger ECHO^®^ Networks had lower rates across several indicators. Over time, it was expected that attendance rates, case presentation numbers, professional discipline diversity, and geographic distribution would continue to mature similarly to more longstanding ECHO^®^ Networks in the sample. Also, despite the low rates of interactivity being measured across the sample, the indicator remains an important characteristic of effective ECHO^®^ Networks [28].

Similar to the concept of network effects, ECHO^®^ Networks’ participation rates and case presentation rates largely grew steadily over a 12-month period. This was likely due to participants having the opportunity to experience what value the ECHO^®^ Networks each had to offer them in terms of support, mentorship, learning, reducing professional isolation, and what they could contribute to the peer-to-peer forum. While these results may seem slow, they should be considered in line with the strong participant experience and satisfaction results covered in Table 3 which highlight consistent positive results across multiple indicators for acceptability and adoption of the new innovation.

### ECHO^®^ network participant experience and impacts

Of particular interest to executive decision-makers and other organizational leaders, this study’s results demonstrated that the implementation and use of the ECHO model™ across this sample played a pivotal role in achieving positive changes in practice for spoke participants. In particular, spoke participants reported positive results in changes to how they approached working with patients/clients/consumers through their procedural tasks and techniques, undertaking assessments, and collecting information to inform case management functions. Where these ECHO^®^ Networks can sustain or increase these outcomes will further reinforce the value that Project ECHO^®^ can offer to innovate and disrupt the status quo to create new value, particularly in workforce development and capacity building.

The results covered above in Table 3 clearly demonstrated that the implementation of Project ECHO^®^ resulted in a broad range of positive outcomes for ECHO^®^ Network participants, which established positive baseline results. It was evident that the use of the ECHO model™ in these settings had achieved solid results for the participants in this sample of ECHO^®^ Networks. While the indicators of overall experience and overall service improvements were not as high, they may increase incrementally as the innovation becomes more business-as-usual similar to other indicators previously mentioned such as rates of case presentations. The experience and satisfaction measures could be administered more regularly to enable implementation teams to identify and respond to local nuances in their specific contexts. To ensure that implementation is as successful as possible, the monitoring the collection of more routine data such as those derived from participants, should be automated through enhancements to the iECHO CRM solution wherever possible. This would enable ECHO^®^ implementation teams to continue to track these trends, address emergent issues and exploit opportunities as their implementation lifecycle evolved. Project ECHO^®^ hub teams should continue to measure these scores locally to contrast with this baseline for quality improvement into the future.

These results add further weight for executive decision-makers to consider when assessing the ongoing investment in Project ECHO^®^ hub operations, given that such positive results were achieved largely within 12–18 months of the new innovation launching. This speed to value for Project ECHO^®^ being implemented as a new innovation across this sample of organizations is noteworthy, given the general prevalence for innovations to fail [13].

### ECHO^®^ network panelist experience and impacts

The results in Table 4 also demonstrate the strong positive experiences and satisfaction of panel experts involved in ECHO^®^ Networks. Similarly, to the participant experience and impacts, hub teams should continue to measure these scores locally to ensure they are maintained over the longer term. These results also reinforce from an internal organizational perspective, that the acceptability and value perception of the innovation was noteworthy. These may be strong indicators to advocate with when implementation teams are seeking ongoing organizational investment in hub operations.

### Research and evaluation resourcing, and data automation

What appeared as a significant gap amongst all but one Project ECHO^®^ hub organization was limited or no access to research, evaluation or librarian disciplines for support. This reinforced the abovementioned challenges with the iECHO CRM solution’s limited automation of data collection and marketing/engagement functions limiting the capability for implementation teams to report on and showcase outcomes. In the case of smaller teams in organizations with less executive decision-maker support or oversight, this gap would likely compound the difficulties faced by implementation teams in demonstrating outcomes to secure more recurrent funding streams for sustainability.

This study identified that the limited access to research and evaluation resourcing was a significant barrier for implementation teams to capture and present their implementation outcomes with scientific rigor to showcase their local impact. This reinforced other research from the United States which explored the recent evidence base and recommended a future research agenda for Project ECHO^®^ at a global level [63]. It is also acknowledged that the challenge of securing access to human resources such as research and evaluation capability may not be unique to Project ECHO^®^ organizations. The competitive landscape that exists around investment in innovation will likely continue implementation teams’ reliance on non-recurrent funding streams over longer periods of time. Despite this, it still enables the continuity of their innovation to demonstrate incremental outcomes and build an evidence base over time. Identifying ways to access research and evaluation capability should be a focus area for implementation teams to prioritize and explore according to their local organizational context. Partnerships with other internal and external stakeholders may support identification of and access to colleagues with research and evaluation skillsets and capacity to collaborate in evaluating local implementation and sustainability efforts.

In addition to research and evaluation resourcing, the current data collection and automation capability with the iECHO CRM was reported to have several limitations. This study’s finding coincided with the ECHO Institute™ announcing in April 2023 that an investment in redeveloping and enhancing the iECHO CRM would occur during late 2023 in a phased roll-out globally. The enhancement would focus on integration and automation features to increase Project ECHO^®^ implementation teams’ convenience and capability to undertake routine data collection, analysis, and reporting. This new capability would address the limitations acknowledged above and allow for the indicators measured in this study to be integrated and scaled for all 1000+ Project ECHO^®^ hub organizations to utilize globally.

### Limitations

#### Study sample

The authors acknowledge that the findings in this sample of 13 organizations are not considered to be representative of all 1000 + organizations which had implemented Project ECHO^®^ at the time of data collection [30]. The timing of data collection for this study (June-September 2022) occurred during the summer holiday period for the Northern Hemisphere– where most Project ECHO^®^ hubs are geographically located, which may have contributed to low participation rates from North American hubs in United States and Canada (4/13, 30.8%). During this time, it was also assumed that the ongoing impacts of the COVID-19 pandemic may have further impacted global response rates. These two facts were anecdotally identified as potential limiting factors for participating organizations to opt-in to the research invitation due to planned and emergent leave and operational hiatuses. This may also explain why hub organizations in Australia appear to be more prominently represented. Future studies should consider scheduling data collection phases to avoid the seasonal hiatuses where practicable. Therefore, these results should be interpreted with caution given the higher response rates represented from Australian organizations.

Despite this, the authors maintain that these findings have still provided unique insights which described the demographic characteristics, implementation variations and factors which were and were not working well within a global sample of organizations which had implemented the ECHO model™, which has not previously been investigated.

In addition, this study presents an in-depth consolidation of findings from various implementations of the ECHO model™ led by motivated employees working within hub organizations across five countries in the last seven years which opted-in to participate in this research. As such, the authors acknowledge that desirability bias cannot be excluded from these findings, given that each organization looked favorably upon the opportunity to participate [64]. These findings may serve as a more diverse and contemporary lens by which to inform other organizational teams of varying sizes, sectors and geographical settings when considering the future implementation planning for, research about, and diffusion of the ECHO model™. These findings are unique in how they establish a baseline for future research to occur. The enhancements to the iECHO CRM solution noted above will also facilitate for larger-scale, automated data collection to inform future research using the indicators measured in this study.

### Scalability to other sectors

While there was almost exclusive participation from healthcare sector organizations in this study, the indicators of success which were measured show potential for universal use in future research across other sectors where Project ECHO^®^ is already in use including education, disability, and beyond.

### Future directions

To build upon the valuable contributions of this baseline study, future longitudinal research should be undertaken to measure these indicators of implementation success across geographies and sectors. Given the timeliness of automation enhancements to the iECHO CRM system used by all ECHO^®^ hub organizations, there is future potential for rich additional insights on where and how implementation success occurs across ECHO^®^ hub organizations globally. Further investigation into measuring peer-to-peer participant advocacy and in-session interactivity within individual ECHO^®^ Networks should also be a priority for future quality improvement research.

## Conclusions

This study makes an empirical contribution to the literature by explaining the process by which implementation teams can measure and report on their implementation success using a universal framework of indicators with real-world examples from the field. It also addresses the gap in the literature to better understand how the ECHO model™ has been implemented and embedded within organizations successfully. Findings from this study offer practical strategies and insights to provide support for executive decision-makers and implementation teams on how they can enhance the successes of their Project ECHO^®^ implementations. These findings will contribute to ongoing knowledge creation of how to successfully embed and leverage innovations like Project ECHO^®^ to enhance service operations and integration across the system [1, 39, 40]. Given the high rates of implementation failure, rising operating costs and competitive fiscal landscape that exists internationally, these learnings will also be valuable to a variety of stakeholder groups. In particular, these findings will support executive decision-makers considering organizational commitment to and financial investment in the adoption of Project ECHO^®^ as a new innovation, and ECHO^®^ Superhub teams as they continue to diffuse the innovation’s adoption in new organizations globally [6, 13, 65].

This study has contributed new evidence to the field by measuring 54 indicators of implementation success, to establish the first known baseline description of Project ECHO^®^ implementations with a global sample. This study offers new international evidence on how these implementations vary across key success indicators, and identified what barriers exist for organizational teams.

Overall, this study highlighted a diversity amongst organizations which have implemented the ECHO model™, with several areas of success highlighted above. These included pre-launch experimentation and expansion, routine Superhub mentorship, internal and external stakeholder engagement, and network alignment to strategic priorities as exemplars that could be replicated in other organizational settings. Similarly, several opportunities for improvement have also been highlighted including securing ongoing investment, measuring peer-to-peer advocacy and interactivity, and the routine use of fidelity assurance tools to support ongoing quality improvement.

This study makes an empirical contribution to the literature by explaining the process by which organizational teams can measure their implementation success using a universal framework of indicators with real-world examples. It also addresses the gap in the literature to better understand how the ECHO model™ has been implemented and embedded within organizations successfully.

Successful implementation should be considered to include a variety of contextual factors specific to the individual organizations in which Project ECHO^®^ is being adopted as a new innovation. This study has illustrated Project ECHO^®^ to be a ‘one model, many implementations’ innovation.

### Electronic supplementary material

Below is the link to the electronic supplementary material.

Supplementary Material 1

Supplementary Material 2

## Data Availability

The datasets used and/or analyzed during the current study will be available from the corresponding author on reasonable request.

## Abbreviations

CME: Continuing Medical Education
CRM: Client Relationship Management system
ECHO: Extension for Community Healthcare Outcomes
FTE: Full-Time Equivalent
HR: Human Resources
