# Supplementary material for: Project ECHO®: a global cross-sectional examination of implementation success
Source: BMC Health Serv Res. 2024 May 3;24:583. doi: 10.1186/s12913-024-10920-5 (PMC11069135; doi:10.1186/s12913-024-10920-5)
Supplement: Supplementary file 1 — Supplementary Material 1 [file 12913_2024_10920_MOESM1_ESM.docx]

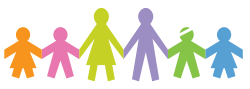

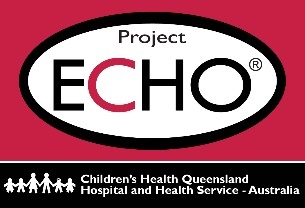

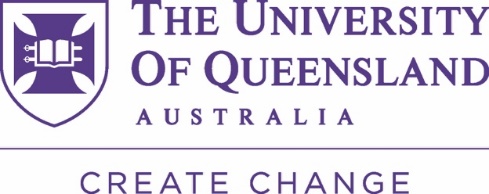


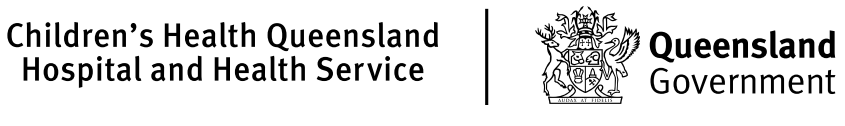


## Additional Information File 1: Indicators of implementation success for Project ECHO®

**File format: Microsoft Word**

**Title of Data:** Indicators of implementation success for Project ECHO®

**Description of Data:** The framework of 54 distinct indicators of Project ECHO® implementation success, categorized across four domains. This file provides recommendations on the measurement phase, data collection point and method of measurement.

This Project ECHO® Implementation Success Framework resource has been adapted from the following publication:

Moss, P., Hartley, N., Newcomb, D., & Russell, T. (2022). Measuring the Success of a Project ECHO Implementation: Results from an International e-Delphi Study. *Global Implementation Research and Applications, 2*(3), 179-194. doi:10.1007/s43477-022-00050-7.

**Context overview:**

There is an increasing global need for organizations in any sector to utilize high-quality telementoring models to support workforce development and mentorship. Project ECHO® is a validated telementoring model that has been adopted globally, with increasing use across multiple sectors beyond healthcare. The below framework presents a consolidated list of success indicators by which organizational teams in any sector can use to assess or benchmark their implementation of Project ECHO®.

The international research study that was conducted to develop this framework used the e-Delphi methodology to identify the indicators of success. The indicators listed in this framework were derived by an international panel of Project ECHO® experts across the healthcare, education, and university sectors. This consolidated framework has been designed to have a practical and scalable application in any organizational or sectoral context to support Project ECHO® teams to successfully measure outcomes associated with their implementation of Project ECHO®.

Being aware of what indicates the successful implementation and how to measure this makes it easier for individuals and teams to exploit and showcase the benefits of the ECHO model™ within their organizational and sectoral context. This will empower teams to gather compelling evidence to convince executive decision-makers and system leaders to financially invest in and organizationally commit to using the ECHO model™. The findings presented in this framework are significant because they are generalisable to any organization/sector implementing Project ECHO®.

The final framework identified 54 distinct indicators across four domains, and provides recommendations on the measurement phase, data collection point and method of measurement:

1. **Spoke participant engagement;**
2. **ECHO® Hub/ECHO® Network design and operation;**
3. **ECHO® Hub team engagement; and**
4. **Local Impact.**

| **#** | **Indicator of Success** | **Recommended Measurement Phase (Pre-Launch, Launch, Growth / Continuous Improvement)** | **Data Collection Point / Stakeholder (Individual Spoke Participant / Panelist, ECHO Network, ECHO Hub, Organization, System)** | **Recommended method/s of Measurement** |
| --- | --- | --- | --- | --- |
| **Domain 1: Spoke Participant Engagement – 14 indicators.**  *Definition: Indicators which measure the number, interactivity and participation experience of individuals who join ECHO® Networks from a variety of spoke locations to connect and learn with panel teams centrally coordinated by the hub.* | | | | |
| D1.1 | Spoke participants attend ECHO Network sessions regularly. | Launch, Growth/ Continuous Improvement | Individual Spoke Participant | iECHO CRM ECHO clinic attendance report: individual participant attendance. |
| D1.2 | Spoke participant diversity (gender, profession, culture, geography) attendance which meets target numbers. | Launch, Growth/ Continuous Improvement | Individual Spoke Participant | iECHO CRM ECHO clinic attendance report: individual participant attendance. |
| D1.3 | Evidence of peer-to-peer testimonials. | Growth/ Continuous Improvement | Individual Spoke Participant | Qualitative documentation of/recordings of testimonials, Spoke Participant Surveys (individuals), Single Session Feedback (polling, surveys), Interviews and Focus Groups. |
| D1.4 | Higher levels of spoke participant experience (enjoyable, collegial, inclusive, non-judgemental). | Growth/ Continuous Improvement | Individual Spoke Participant | Spoke Participant Surveys (individuals), Single Session Feedback (polling, surveys), Interviews and Focus Groups. |
| D1.5 | Number of spoke participants who present cases for discussion. | Growth/ Continuous Improvement | Individual Spoke Participant | iECHO CRM participant report, iECHO CRM ECHO clinic report. |
| D1.6 | Number of ECHO sessions where spoke participants present cases from their local context. | Growth/ Continuous Improvement | ECHO Network | iECHO CRM participant report, iECHO CRM ECHO clinic report. |
| D1.7 | Higher levels of spoke participant safety and comfort in volunteering to present cases from their own context as a learning opportunity within the ECHO Network. | Growth/ Continuous Improvement | Individual Spoke Participant | Spoke Participant Surveys (individuals), Single Session Feedback (polling, surveys), Interviews and Focus Groups, iECHO CRM ECHO clinic report. |
| D1.8 | Higher levels of spoke participant satisfaction with didactic content, panel expert(s) representation/hub team support. | Growth/ Continuous Improvement | Individual Spoke Participant | Spoke Participant Surveys (individuals), Single Session Feedback (polling, surveys), Interviews and Focus Groups, iECHO CRM ECHO clinic report. |
| D1.9 | Higher levels of spoke participant satisfaction with learning/advice/support gained from case presentation and discussion (applies to individual case presenter, as well as other spoke participants learning from the case) and recommendations. | Growth/ Continuous Improvement | Individual Spoke Participant | Spoke Participant Surveys (individuals), Single Session Feedback (polling, surveys), Interviews and Focus Groups. |
| D1.10 | Number of spoke participants who represent cases. | Launch, Growth/ Continuous Improvement | Individual Spoke Participant | iECHO CRM participant report, iECHO CRM ECHO clinic report. |
| D1.11 | Higher levels of spoke participant satisfaction with the opportunity to contribute to the dialogue, ask questions, make recommendations whether verbally or non-verbally. | Growth/ Continuous Improvement | Individual Spoke Participant | Spoke Participant Surveys (individuals), Single Session Feedback (polling, surveys), Interviews and Focus Groups. |
| D1.12 | High reported levels of spoke participants self-reporting that they feel safe, supported, and welcomed at ECHO Network sessions. | Growth/ Continuous Improvement | Individual Spoke Participant, ECHO Network | Spoke Participant Surveys (individuals), Single Session Feedback (polling, surveys), Interviews and Focus Groups, iECHO CRM ECHO clinic report. |
| D1.13 | Measurable increase in spoke participants who contribute to the discussion verbally or via chat. | Growth/ Continuous Improvement | ECHO Network | ECHO Scorecard. |
| D1.14 | Evidence of spoke participants inviting colleagues to attend ECHO Network sessions to co-present case presentations. | Growth/ Continuous Improvement | Individual Spoke Participant, ECHO Network | iECHO CRM participant report, iECHO CRM ECHO clinic report. |
| **Domain 2: ECHO® Hub/ECHO® Network design and operation – 23 indicators.**  *Definition: Indicators which measure the design and operation of an organization’s ECHO® Hub, and/or individual ECHO® Networks.* | | | | |
| D2.1 | Evidence of the ECHO Network's co-design occurred with prospective participants, consumers, system managers, and subject matter experts. | Pre-Launch, Growth/ Continuous Improvement | ECHO Network | Review of Implementation plan, Evaluation plan, Learning Needs Assessment results. |
| D2.2 | Number of discrete stakeholders involved in the co-design of the ECHO Network. | Pre-Launch, Growth/ Continuous Improvement | ECHO Network | Review of Implementation plan, Evaluation plan, Learning Needs Assessment results. |
| D2.3 | Demonstrated alignment to local, state, federal priorities, and associated quality/funding metrics. | Pre-Launch, Growth/ Continuous Improvement | ECHO Network, ECHO Hub, Organization, System | Review of Implementation plan, Evaluation plan, Learning Needs Assessment results, and ECHO Network funding sources. |
| D2.4 | Evidence that the ECHO Network delivers on the findings of the learning needs assessment. | Growth/ Continuous Improvement | ECHO Network, ECHO Hub. | Review of Implementation plan, Evaluation plan, Learning Needs Assessment results, Spoke Participant Surveys (individuals), Single Session Feedback (polling, surveys), Interviews and Focus Groups. |
| D2.5 | Measurable increase in levels of interactivity amongst spoke participants and panelists during sessions (on camera, chat, verbal, non-verbal, volunteering to present cases). | Growth/ Continuous Improvement | ECHO Network - Spoke Participants, Panelists | ECHO Scorecard. |
| D2.6 | Frequency/ regularity of sessions - sessions are held routinely. | Launch, Growth/ Continuous Improvement | ECHO Network | iECHO CRM participant report, iECHO CRM ECHO clinic report. |
| D2.7 | Higher levels of balance in dialogue contributed by panelists vs spoke participants, demonstrating spokes are contributing at least 50% of the talking. | Growth/ Continuous Improvement | ECHO Network | ECHO Scorecard. |
| D2.8 | Number of ECHO sessions including a participant case. | Launch, Growth/ Continuous Improvement | ECHO Network | iECHO CRM participant report, iECHO CRM ECHO clinic report. |
| D2.9 | High levels of ECHO sessions being a non-hierarchical, professional forum for knowledge sharing is fostered by panelists. | Launch, Growth/ Continuous Improvement | Individual ECHO Network Panelist, ECHO Network | ECHO Scorecard, Spoke Participant Surveys (individuals), Single Session Feedback (polling, surveys), Interviews and Focus Groups. |
| D2.10 | Evidence of streamlined hub operational and logistical processes that optimise the delivery of ECHO sessions. | Pre-Launch, Growth/ Continuous Improvement | ECHO Network, ECHO Hub | ECHO Scorecard, iECHO CRM participant report, iECHO CRM ECHO clinic report, evidence of localised policies, procedures, manuals for ECHO hub operations. |
| D2.11 | Evidence that the ECHO Panel adheres to the Anatomy of an ECHO for fidelity assurance. | Launch, Growth/ Continuous Improvement | Individual ECHO Network Panelist, ECHO Network | ECHO Scorecard, Spoke Participant Surveys (individuals), Single Session Feedback (polling, surveys), Interviews and Focus Groups. |
| D2.12 | Evidence of ECHO hub teams undertaking learner needs assessment, implementation planning, evaluation planning, panel expertise onboarding, and 2 mock ECHO sessions prior to launching a ECHO Network. | Pre-Launch. | Individual ECHO Network Panelist, ECHO Hub | Review of Implementation plan, Evaluation plan, Learning Needs Assessment results, Interviews and Focus Groups, mock and post-launch ECHO Scorecards. |
| D2.13 | Evidence of hub team attracting sufficient funding to fulfil implementation/ hub management/ replication functions sustainably. | Pre-Launch, Launch, Growth/ Continuous Improvement. | ECHO Hub | Organizational cost centre/financial reports. |
| D2.14 | Evidence of hub team managing operations within budget constraints of the organization. | Launch, Growth/ Continuous Improvement | ECHO Hub | Organizational cost centre/financial reports. |
| D2.15 | Evidence of ECHO hub leadership role(s) and clear organizational governance oversight of ECHO hub team structure are present. | Pre-Launch, Launch, Growth/ Continuous Improvement. | ECHO Hub | ECHO team role descriptions, organizational structure, organization's operational and/or strategic plans. |
| D2.16 | Evidence of an interprofessional and diverse hub team. | Pre-Launch, Launch, Growth/ Continuous Improvement. | ECHO Hub | ECHO team role descriptions, organizational structure. |
| D2.17 | Evidence of communication systems/processes developed for routine engagement with stakeholders outside of ECHO sessions. | Launch, Growth/ Continuous Improvement | Individual ECHO Network Panelist, ECHO Hub | Correspondence records, mailing lists, templates/CRM for distribution of didactic resources, reference lists, journal articles, podcasts, and other resources. |
| D2.18 | Evidence of hub team’s development and dissemination of marketing materials to increase awareness of and attraction to their ECHO operations. | Pre-Launch, Launch, Growth/ Continuous Improvement. | Individual ECHO Network Panelist, ECHO Hub | Media analytics dashboards that can be tailored/shared widely across multiple stakeholder audiences as appropriate including content attesting to the quality/credibility of organizational hub team/panelists. Examples would include succinct and engaging marketing materials to increase awareness of and attraction to ECHO activities and be tailored/shared widely across multiple audiences. |
| D2.19 | Evidence of panelists and spoke participants advocating via word of mouth, peer-to-peer, personal/ professional network communication/ recommendations about joining ECHO network(s). | Pre-Launch, Launch, Growth/ Continuous Improvement. | Individual Spoke Participant, Individual ECHO Network Panelist, ECHO Network | Qualitative documentation of/recordings of testimonials, Spoke Participant Surveys (individuals), Single Session Feedback (polling, surveys), Interviews and Focus Groups. |
| D2.20 | Evidence of hub stakeholders (champion, facilitator, panelists, coordinator) completing ECHO Immersion training provided by a designated Superhub prior to launch. | Pre-Launch. | Individual ECHO Network Panelist, ECHO Hub | Immersion attendance records. |
| D2.21 | Evidence of ECHO hub team engaging with Superhub for post-Immersion partner liaison support and mentorship. | Pre-Launch, Launch, Growth/ Continuous Improvement. | Individual ECHO Network Panelist, ECHO Hub | iECHO/Salesforce CRM reports (partner liaison, technical assistance). |
| D2.22 | Evidence of hub teams having data collection processes to ensure all pertinent data is collected and evaluated in a reliable way. | Pre-Launch, Launch, Growth/ Continuous Improvement. | ECHO Network Panel, ECHO Hub | ECHO Scorecard records, iECHO CRM reports, evidence of localised protocols for ECHO hub data collection and evaluation. |
| D2.23 | Evidence of executive/ leadership support - where ECHO activities strategically align to organizational priorities, funding/investment decision-making. | Pre-Launch, Launch, Growth/ Continuous Improvement. | ECHO Hub, Organization | Qualitative and quantitative documentation of/recordings of executive/leadership endorsement/advocacy for ECHO, reference points in organizational strategies, policies, plans, financial cost centre reports. |
| **Domain 3: ECHO® Hub team engagement – 5 indicators.**  *Definition: Indicators which measure the number, interactivity and participation experience of individuals who facilitate and manage ECHO® Hub functions.* | | | | |
| D3.1 | High levels of panelist experience and satisfaction (enjoyable, high value, time efficient). | Growth/ Continuous Improvement | Individual ECHO Panelist, ECHO Network | Panelist Surveys (individuals), Single Session Feedback (polling, surveys), Interviews and Focus Groups. |
| D3.2 | Evidence of relevant stakeholders having a clear understanding of the ECHO model, its theoretical and practical application, and potential benefits. | Launch, Growth/ Continuous Improvement | Individual ECHO Network Panelist, ECHO Hub, Organization | Review of Implementation plan, Evaluation plan, Learning Needs Assessment results, Interviews and Focus Groups. |
| D3.3 | High levels of strong and organised facilitation role/function, panel cohesion and satisfaction during ECHO Network sessions. | Launch, Growth/ Continuous Improvement | Individual ECHO Network Panelist, ECHO Network | ECHO Scorecard, evidence of localised policies, procedures, manuals for ECHO hub operations. |
| D3.4 | Demonstrated ability to recruit and retain Champion, Facilitator, Panelists with the right qualification, skills, expertise, lived experience, ability to present well and make spoke participants feel comfortable. | Launch, Growth/ Continuous Improvement | Individual ECHO Network Panelist, ECHO Network | ECHO Scorecard, ECHO team role descriptions. |
| D3.5 | High levels of panel facilitator and panelist satisfaction with learning/ advice/ support contributed to/ arising from case presentation/s (applies to panel contributions, individual case presenter, as well as other spoke participants' learning and contribution to recommendations for the case/s). | Launch, Growth/ Continuous Improvement | Individual ECHO Network Panelist, ECHO Network | Panelist Surveys (individuals), Single Session Feedback (polling, surveys), Interviews and Focus Groups. |
| **Domain 4: Local Impact – 12 indicators.**  *Definition: Indicators which measure the increase or improvement in workforce development, capacity, system integration and efficiency.* | | | | |
| D4.1 | Measurable increase in spoke participant's confidence to manage cases locally. | Pre-Launch, Growth/ Continuous Improvement | Individual Spoke Participant | Spoke Participant Surveys (individuals), Single Session Feedback (polling, surveys), Interviews and Focus Groups. |
| D4.2 | Measurable increase in spoke participant's competence to manage cases locally. | Pre-Launch, Growth/ Continuous Improvement | Individual Spoke Participant | Spoke Participant Surveys (individuals), Single Session Feedback (polling, surveys), Interviews and Focus Groups. |
| D4.3 | Measurable increase in spoke participant's knowledge/skills to manage cases locally. | Pre-Launch, Growth/ Continuous Improvement | Individual Spoke Participant | Spoke Participant Surveys (individuals), Single Session Feedback (polling, surveys), Interviews and Focus Groups. |
| D4.4 | Measurable increase in spoke participant's capacity to manage cases locally. | Pre-Launch, Growth/ Continuous Improvement | Individual Spoke Participant | Spoke Participant Surveys (individuals), Single Session Feedback (polling, surveys), Interviews and Focus Groups. |
| D4.5 | Measurable increase in spoke participant self-reported change in experience to become a local expert to whom colleagues in their community/proximity refer to and collaborate with for support on cases. | Pre-Launch, Growth/ Continuous Improvement | Individual Spoke Participant | Spoke Participant Surveys (individuals), Single Session Feedback (polling, surveys), Interviews and Focus Groups. |
| D4.6 | Spoke participants applying of at least one change in their practice due to their participation in ECHO Networks. | Growth/ Continuous Improvement | Individual Spoke Participant | Spoke Participant Surveys (individuals), Single Session Feedback (polling, surveys), Interviews and Focus Groups. |
| D4.7 | Measurable increase in spoke participant self-efficacy. | Pre-Launch, Growth/ Continuous Improvement | Individual Spoke Participant | Spoke Participant Surveys (individuals), Single Session Feedback (polling, surveys), Interviews and Focus Groups. |
| D4.8 | Measurable reduction in spoke participant's sense of professional isolation. | Pre-Launch, Growth/ Continuous Improvement | Individual Spoke Participant | Spoke Participant Surveys (individuals), Single Session Feedback (polling, surveys), Interviews and Focus Groups. |
| D4.9 | Measurable increase in spoke participant's joy of work. | Pre-Launch, Growth/ Continuous Improvement | Individual Spoke Participant | Spoke Participant Surveys (individuals), Single Session Feedback (polling, surveys), Interviews and Focus Groups. |
| D4.10 | Higher spoke participant reported positive changes in knowledge-sharing relationships between colleagues locally. | Growth/ Continuous Improvement | Individual Spoke Participant, ECHO Network | Spoke Participant Surveys (individuals), Single Session Feedback (polling, surveys), Interviews and Focus Groups. |
| D4.11 | Improvements in service utilisation, service wait times, distance travelled to access services by patients/consumers/ clients. | Launch, Growth/ Continuous Improvement | Individual consumer, Individual ECHO Spoke Participant, ECHO Network, ECHO Hub, Organization, System | ECHO case presentation and patient/client record audits, postcode mapping, economic modelling, and analysis. |
| D4.12 | Improvements in spoke participant’s professional relationships, access to specialist services, referral pathways, informed decision-making, peer-to-peer supports outside of ECHO sessions which impact their patient/client care/service provision/professional isolation. | Launch, Growth/ Continuous Improvement | Individual Spoke Participant | Qualitative documentation of/recordings of testimonials, Spoke Participant Surveys (individuals), Single Session Feedback (polling, surveys), Interviews and Focus Groups. Social Network Analyses. |
