# Supplementary material for: Project ECHO®: a global cross-sectional examination of implementation success
Source: BMC Health Serv Res. 2024 May 3;24:583. doi: 10.1186/s12913-024-10920-5 (PMC11069135; doi:10.1186/s12913-024-10920-5)
Supplement: Supplementary file 2 — Supplementary Material 2 [file 12913_2024_10920_MOESM2_ESM.docx]

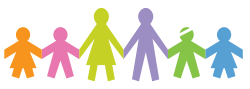

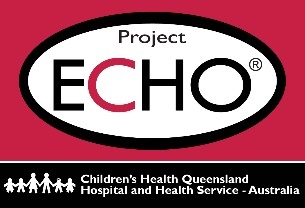

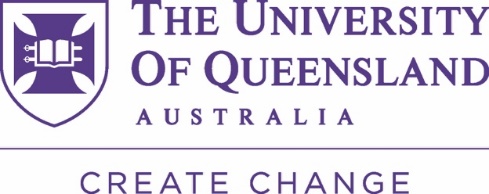


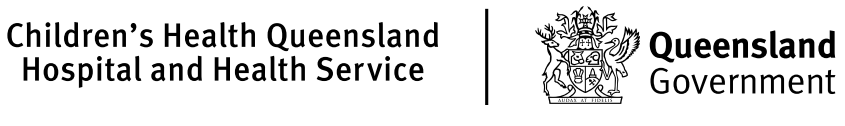


## Additional Information File 2: Data Collection Tool

**File format:** Microsoft Word

**Title of Data:** Data Collection Tool

**Description of Data:** Complete 78-question Part A and Part B survey tool for data collection against the 54 indicators of Project ECHO® implementation success.

Part A

**Welcome**
**Part A: A cross-sectional, international state of play for Project ECHO® Implementations:**
Welcome to the study! This survey portal will be open and accepting submissions until **Sunday 30 October 2022.**

Please complete this section as a **single submission** for your **overall ECHO Hub**. You **DO NOT need to complete Part A more than once**.

It is recommended that your ECHO Network Coordinators collate network-specific data for individual **Part B submissions** and administer the single session polls for each of the ECHO networks they coordinate for consistency. ECHO hub leads, facilitators and/or champions can approve the data for submission to the investigators.

You will be required to **submit your data in the Qualtrics online survey**. However, prior to this for convenience, you can compile your data in this fillable Survey Guide document as it is collected. This data can then be easily transferred to the final survey once you have all data collected. As a result of participating, you will receive a compiled report for your overall ECHO hub and each ECHO network along with aggregated benchmarking data from other participating sites at the end of this study.

 **Human Research Ethics Summary and Study Information:**

This study has ethics approval through the HREC committee at Children’s Health Queensland Hospital and Health Service, and The University of Queensland (HREC approvals no: HREC/22/QCHQ/86967, 2022/HE001044 and SSA-2022-QCHQ-86967). This study will examine the various implementations of Project ECHO within organisations worldwide from across multiple sectors to determine the current state of play using an implementation success framework. This survey portal will be open and accepting submissions until **Sunday 30 October 2022.**

The Children’s Health Queensland ECHO Superhub in partnership with the University of Queensland are inviting all members of the MetaECHO community to participate in an international state of play research study.

**Investigators:**

Perrin Moss (CHQ, and PhD Candidate, UQ), Dr Dana Newcomb (CHQ), Dr Nicole Hartley, and Professor Trevor Russell (UQ).

**This study aims to:**

- To understand how implementations of Project ECHO compare across international and sectoral contexts;
- To understand where implementations of Project ECHO can be improved across international and sectoral contexts;
- To understand what supports are required by organisational teams implementing Project ECHO to overcome barriers; and
- To gauge what the current international state of play is for Project ECHO implementations.

**Benefits to your ECHO hub:** This research will provide ECHO practitioners, executives, decision-makers, and other partners collaborating with participating ECHO hubs with a personalised report of their hub’s local state of play, contrasted with aggregated global results.

**What does your participation look like?** You will be asked to complete a two-part online survey that will take less than one hour to complete related to implementation success. There is approximately 2-3 hours of data collation depending on the number of ECHO Networks you submit data for that your ECHO hub may need to collect as a once-off in single session polls if this information is not routinely collected by your hub over and above usual iECHO data reporting. You may also be invited to participate in an interview of up to one hour after the online survey has been completed. The deadline for participating ECHO hubs to submit their data is **30 October 2022**.

Fortnightly email reminders will be circulated to ensure opportunity to participate is afforded to as many participating sites.

**Online drop-in information sessions:** You will have the opportunity to attend virtual drop-in information sessions that will be hosted by the Principal Investigator via Zoom. The purpose of these sessions will be to discuss the approach to follow when completing your data collection requirements for Part B (ECHO Network specific data). You will be provided with real-time support to clarify what is required to participate in the study. The timing of the drop-in sessions will be offered to suit the local time zones of participating ECHO hub sites.

**What do you get out of participating?** As a result of your participation, your ECHO hub will receive a personalised report analysing your local state of play, contrasted with aggregated global results to enable you to benchmark your implementation and subsequent performance. This research will also provide recommendations for ECHO Superhubs, as training and support centres for new and existing hub organisations, on quality improvement and implementation support resources that may enhance ECHO hub teams to address barriers that impact implementing the model successfully.

**Why have I been invited to participate in this study?** You have been invited to participate in this study because you occupy a role within an existing Project ECHO hub organisation.

**What if I don’t want to take part in this study, or if I want to withdraw later?** Your decision to take part in this study is entirely voluntary. Your relationship with the Principal Investigator, CHQ and UQ, will not be affected by the decision you make to participate or not. You are free to withdraw from the study at any time without having to give a reason. There are no consequences for withdrawal from this study. If you decide to stop participating, your data will be used up to the point at which you decide to withdraw.

**How will my privacy be protected?** All information provided by you in the study will be kept confidential. Any information you give us will be stored securely. When we store it, we will give it a code rather than label it with your name/organisation. Only people within the research team will have access to this information. Any published data will not reveal your identity. Following interviews, you will be offered the opportunity to review your hub’s personalised report. Our reports will include a summary of all the information gathered from all participants in this study but will not be linked to any identifying information or personal details. All data obtained in this study will be kept secure during the study and for a period of five years following the completion of the study. After this time all information and recordings will be destroyed.

**Who should I contact if I have concerns about the conduct of this study?** This study adheres to the guidelines of the ethical review process of The University of Queensland and the National Statement on Ethical Conduct in Human Research. The Children’s Health Queensland Hospital and Health Service Human Research Ethics Committee (HREC) has approved this study. If you have any concerns and/or complaints about the project, the way it is being conducted or your rights as a research participant and would like to speak to someone independent of the project, please contact the HREC Co-ordinator on: +617 3069 7002 or email [CHQETHICS@health.qld.gov.au](mailto:CHQETHICS@health.qld.gov.au).
**Please click the arrow in the below right corner to commence your Part A submission.**

**Informed Consent:**
 I have read the participant information sheet and by clicking accept below and completing the survey, I am confirming my consent to participate. I hereby consent to participate in research activities implementing Project ECHO within an organisational context.

- - I have been given clear information, both written and verbal, about the study, and understand what is required of me:
  - I understand that my participation is voluntary.
  - I may refuse to answer any question and I remain free to withdraw from the study at any time without explanation.
  - I understand that if I decide to stop participating, my data will continue to be used up to the point where I decide to stop participating.
  - I am aware that interviews will be audio recorded and transcribed.
  - I am aware that survey responses will be recorded and analyzed.
  - I understand that all information from the sessions will remain confidential to the research team and that information will be securely stored with all identifying information removed.
  - I understand that the information that I provide will not be described or portrayed in ways that may identify me in any report on the study.
  - I am aware that I may ask any further questions about the research study at any time.
  - I am aware that I will have the opportunity to review and comment on transcriptions and observational field notes.
     The data input time should take approximately 60 minutes to complete Part A and Part B after the requisite data is collated for your hub's activities.

Data collation times may vary by hub depending on the number of ECHO Networks you elect to submit data for.

By clicking the next button below, you are consenting to participate in this study. You may withdraw from this study at any time without any penalty to you. Should you withdraw at any time, your data that has been provided will be analyzed up to the point at which you withdraw.

- Yes, I consent

If you wish to receive research summaries and publication information following the completion of this project, please choose from the below options. You will be asked to confirm your email address in the next section. You will receive an email from the Principal Investigator with the details of this study’s outcomes.

- Yes, please.
- No, thank you.

0.3 If you wish to receive research summaries and publication information following the completion of this project, please enter your preferred email address below.
You will receive an email from the Principal Investigator with the details of this study’s outcomes.

- Email address __________________________________________________

**Please click the arrow in the below right corner to commence your Part A submission.**

**1** Please enter your name (individual respondent)

________________________________________________________________

**2** Please enter your email address (individual)

- Email address __________________________________________________

**3** Please enter your ECHO Hub organisation name (in full)

________________________________________________________________

**4** Please enter the country in which your ECHO Hub is based?

________________________________________________________________

**5** Please select which option best fits the role you occupy within your ECHO Hub

- Executive leader/Decision-Maker
- Discipline leader/ECHO champion
- Program/Project Manager
- ECHO Network Facilitator
- ECHO Network Coordinator
- ECHO IT Support role
- Other - please specify __________________________________________________

**6** Please indicate all the relevant roles you have collaborated with to provide your Hub's response to this survey:

- Executive leader/Decision-Maker
- Discipline leader/ECHO champion
- Program/Project Manager
- ECHO Network Facilitator
- ECHO Network Coordinator
- ECHO IT Support role
- Other - please specify __________________________________________________

**7** Please select the **best-fit** sector in which your ECHO hub organisation

- Advocacy
- Agriculture
- Business
- Civics and government
- Climate change
- Consultancy (private sector)
- Domestic and family violence
- Education (primary/middle/secondary school)
- Education (non-school setting)
- Emergency response / Disaster management
- Healthcare - public health
- Healthcare - primary health
- Healthcare - secondary health
- Healthcare - tertiary/quaternary health
- Information technology
- Law enforcement / Crime
- Legal / Insurance / Investigations
- Mining / Resources
- Professional body (representative/training, not university sector)
- Quality improvement
- Research (including clinical trials)
- Science
- Social welfare
- Veterinary science
- University - research/academic, non-service delivery
- Others - please specify __________________________________________________

**8** Please select **as many** relevant sectors in which your ECHO hub activities **have interfaces** (cross sector partnerships for panel expertise, spoke participants)

- Advocacy
- Agriculture
- Business
- Civics and government
- Climate change
- Consultancy (private sector)
- Domestic and family violence
- Education (primary/middle/secondary school)
- Education (non-school setting)
- Emergency response / Disaster management
- Healthcare - public health
- Healthcare - primary health
- Healthcare - secondary health
- Healthcare - tertiary/quaternary health
- Information technology
- Law enforcement / Crime
- Legal / Insurance / Investigations
- Mining / Resources
- Professional body (representative/training, not university sector)
- Quality improvement
- Research (including clinical trials)
- Science
- Social welfare
- Veterinary science
- University - research/academic, non-service delivery
- Others - please specify __________________________________________________

**9** Please confirm what Replication Phase you consider your ECHO Hub to currently be in:

- **Pre-Launch** (completed Immersion, but not launched your pilot network’s first session)
- **Launch** (completed Immersion, and launched your pilot network with at least one session)
- **Growth/Continuous Improvement** (completed Immersion, launched your pilot network, +/- additional networks, and continue to operate as an ECHO hub)

**10** Please enter the month and year in which your organisation first completed ECHO Immersion training

- Date DD/MM/YYYY __________________________________________________

**11** For your ECHO hub, is there recorded evidence of hub stakeholders that have completed the formal ECHO Immersion training?

- No
- Yes - please confirm the number of staff completed Immersion pre-implementation __________________________________________________
- Yes - please confirm the number of staff completed Immersion post-implementation __________________________________________________

**12** For your ECHO hub, approximately how many times did you engage with the Superhub that delivered your Immersion training for advice and technical assistance in the 12 months following Immersion (email enquiries, Zoom meetings, phone calls, etc)?

- Number of engagements __________________________________________________

**13** Please enter the month and year in which your organisation first launched your pilot ECHO Network

- Date DD/MM/YYYY __________________________________________________

**14** Please enter the **total number** of unique ECHO Networks your organisation has **launched** thus far

- Number __________________________________________________

**15** Please enter the **total number** of **current** ECHO Networks your organisation is running

- Number __________________________________________________

**16** Please enter the number of full-time equivalent staff employed within your ECHO Hub (i.e.: 3.0 FTE)

- Number __________________________________________________

**17** Please enter the total number of individual staff/partners who collaborate with your hub's operational functions (i.e.: Champion/s, Facilitator/s Panelists, Coordinators, Researchers, Volunteers, etc.)

- Number __________________________________________________

**18** For your ECHO hub, is there documented operational and logistical processes that can be reviewed as required to optimise the delivery of the hub and network functions? From the list below, please specify all that your hub uses:

- None
- Work instructions
- Procedures
- Templates
- Checklists
- teleECHO scorecard
- Anatomy of an ECHO
- Agendas
- Panel role cards
- Others (please specify) __________________________________________________

**19** For your ECHO hub, please select all relevant types of funding that was attracted to undertake the ECHO **implementation** within your organisation:

- None
- Organisational funding - temporary
- Organisational funding - recurrent
- Competitive grant
- Commissioning/contracted funding
- Philanthropic grant
- Others (please specify) _________________________________________________

**20** For your ECHO hub, please select all relevant types of funding that has been attracted to facilitate **ongoing ECHO hub management** functions:

- None
- Organisational funding - temporary
- Organisational funding - recurrent
- Competitive grant(s)
- Commissioning/contracted funding
- Philanthropic grant(s)
- Other (please specify) __________________________________________________

**21** For your ECHO hub, please select all relevant options from the below list that could demonstrate how the fixed and temporary costs associated with the hub management/operations have been integrated within your organisational context?

- None
- Organisational finance statements/reports
- ECHO specific cost center statements/reports
- Others (please specify) __________________________________________________

**22** For your ECHO hub, please select all relevant types of governance processes for ECHO activities that are documented within your organisation:

- None
- Decision-making framework
- Human Resource/Professional line management procedures
- Financial management/delegations
- Organisational leadership role oversight
- Others (please specify) __________________________________________________

**23** For your ECHO hub, please list the number of different professional disciplines involved in your operations (medical, nursing, teacher, scientist, administration, etc)?

- Number __________________________________________________

**24** For your ECHO hub, please list the types of different professional disciplines involved in your operations (medical, nursing, teacher, scientist, administration, etc)?

________________________________________________________________

________________________________________________________________

________________________________________________________________

________________________________________________________________

________________________________________________________________

**25** For your ECHO hub, please select all relevant marketing processes and activities that can be evidenced to increase awareness of and attraction to your ECHO operations

- Marketing strategy/plan
- Information flyers
- Website
- Social Media posts (LinkedIn, Facebook, Twitter, other)
- Formal media releases
- Others (please specify) __________________________________________________

**26** For your ECHO hub, is there evidence of panelists and spoke participants advocating (via word of mouth, peer-to-peer, personal/professional network communication/ recommendations) about joining any of the teleECHO network(s) your hub offers?

- Yes, if so, what percentage were positive? __________________________________________________
- No

**27** For your ECHO hub, please select from the below list any data collection processes that are in place to ensure all pertinent data is collected and evaluated in a reliable way?

- Spoke participant registration lists
- Client relationship management database entries (iECHO)
- Client relationship management database entries (other than iECHO - please specify) __________________________________________________
- TeleECHO clinic templated documents
- Case presentation templates
- Consent protocols
- Research Ethics Approvals (Institutional Review Board/Human Research Ethics Committee or equivalent)
- Others (please specify) __________________________________________________

**28** For your ECHO hub, please select all relevant executive/leadership support evidence that exists for using ECHO within your organisation (where ECHO activities strategically align to organisational priorities, funding/investment decision-making, etc)

- None
- Email correspondence
- Briefing Notes or other official correspondence including letters of endorsement
- Project documentation (approved project plans, status reports, etc.)
- Legal documentation (ECHO Partnership Agreements, Memorandums of Understanding, Service Agreements for funding, resources or collaboration, etc.)
- Others (please specify) __________________________________________________

**29** *Hub members to complete a* ***single session poll****:*
 How would you rate your ECHO hub team's understanding of the ECHO model, its theoretical and practical application, and potential benefits in your context?
Please use a 10-point Likert scale to poll the following responses:

- What is your hub's overall (average) rating out of 10 __________________________________________________
- What percentage of your hub team scored 5/10 or above __________________________________________________
- Do you routinely measure this?__________________________________________________
- If yes, have you observed an increase over time? Increase/Decrease/Stable __________________________________________________
- If you have experienced an increase, over what time period (months)? __________________________________________________
- Do you routinely measure this with another tool? If so, what tool? __________________________________________________
- If you do not collect this data, please explain why __________________________________________________

Thank you
We appreciate the time you have taken to participate. 
**Please click the arrow below to finalise your Part A submission.**
**You can proceed to complete Part B by copying the below link into your browser, or via the Survey Guide:**
**** Qualtrics Link for Part B submission embedded here ****If you have any questions, please contact [Perrin.Moss@health.qld.gov.au](mailto:Perrin.Moss@health.qld.gov.au) so a Zoom information session can be scheduled at a convenient time for you.


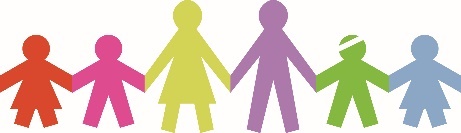

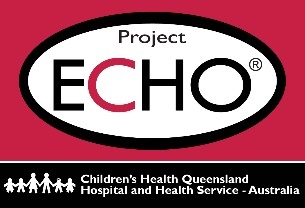

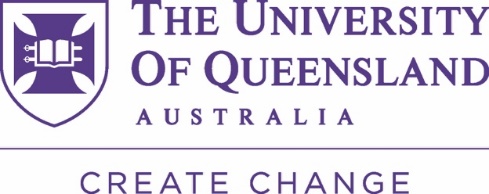


Part B

Welcome **Part B: A cross-sectional, international state of play for Project ECHO® Implementations:**

Welcome back for Part B of the study! This survey portal will be open and accepting submissions until **Sunday 30 October 2022**.

In order to generate the individualised report for your ECHO Hub, please complete this section as a **single submission** for **EACH ECHO Network** your hub has launched. Please also include networks that have concluded or paused with the historical data you have access to.

**You DO NOT need to recomplete Part A.**

It is recommended that your **ECHO Network Coordinators** are requested to collate network-specific data for this submission and administer the single session polls for the networks they coordinate for consistency.

**ECHO hub leads, facilitators and/or champions** can review and clear the data for submission to the investigators.

This can be compiled in the fillable Survey Guide document, prior to submitting the data in the Qualtrics online survey. As a result, you will receive a compiled report for your hub along with benchmarking data from other participating sites at the end of this study.

For Part B, please submit data for as many ECHO Networks as you can for this once-off data collection and analysis phase. This will provide your hub with a more comprehensive network-specific segment in your final report that you can use to review and benchmark against aggregated hub data from all other respondents.

1. Please note there will be questions throughout this part of the survey which require data from your historical and latest ECHO sessions for comparison;
2. For questions with a single session poll, please administer this at your next possible ECHO session by inputting the question in the Zoom polling function; and
3. You can then take a screenshot of the results in the live session to enter afterwards into your fillable Survey Guide and then input into this Qualtrics online survey.
    
   Please click the arrow in the below right corner to commence your Part B single submission.

**1** Please enter your name (individual respondent)

________________________________________________________________

**2** Please enter your individual email address for data linkage purposes across Part A and B submissions

________________________________________________________________

**3** Please enter your ECHO Hub organisation name in full

________________________________________________________________

**4** Please enter the name of this ECHO Network in full

________________________________________________________________

**5** Please enter the focus area/objective of this ECHO Network

________________________________________________________________

**6** When did this ECHO Network first launch? Refer to your iECHO record for the date.

- First Session Date DD/MM/YYYY __________________________________________________

**7** What type of ECHO Network is this? Please select a best-fit response from the below options

- Cohort
- Continuous
- Drop-In
- Hybrid - please specify __________________________________________________

**8** How frequently do you run sessions for this ECHO Network? Please select a best-fit response from the below options

- Daily
- Weekly
- Fortnightly
- Monthly
- Quarterly
- Other - please specify __________________________________________________

**9** Is this ECHO Network running at present?

- Yes, currently running
- Paused - please enter the date of the last session __________________________________________________
- Concluded - please enter the date of the last session __________________________________________________

**10** **Attendance:** For this ECHO Network, please confirm the following metrics:

- What is the total number of spoke participants who have attended to date? (Number of spoke participants since your launch session) __________________________________________________
- What is the total number of spoke participants who have attended in the 12 months preceding your last session? __________________________________________________
- What is the average number of spoke participants who attended per session, since launching? __________________________________________________
- What is the proportion of attendees participating in 80% or more sessions, since launching? __________________________________________________

**11** **Spoke Participant Diversity:** For this ECHO Network, please confirm the following metrics:

- If you do not collect this data, please explain why __________________________________________________
- Gender: Female/Male/Non-binary/Transgender/Intersex/Free Text/I prefer not to say (Please list all that apply) __________________________________________________
- Profession (Please list all that apply) __________________________________________________
- Race/Ethnic Groups (Please list all that apply) __________________________________________________
- Geography (Metro, Regional, Rural and Remote) __________________________________________________

**12** **Point in Time Confirmation:** For this question, please confirm the total number of spoke participants and panel members for ECHO Network who participated in the single session poll questions at your most recent ECHO Network session.  These will be the totals you will refer to for consistency in all single session polling questions throughout this survey:

- Individual spoke participants total (number) __________________________________________________
- Individual panelist team members, including facilitator, panelists, network coordinator, IT, etc. (number) __________________________________________________

**13** **Peer Testimonials:**For this ECHO network, do you have evidence that peer-to-peer testimonials are occurring?

- Yes/No __________________________________________________
- If yes, what percentage of testimonials are positive? __________________________________________________
- If you do not collect this data, please explain why __________________________________________________

**14** **Spoke Participant Experience:** Administer this question for **Spoke Participants** to complete a **single session poll**:
 How would you rate your experience as a spoke participant in this ECHO Network? (Scale: 0 = Not Very Enjoyable at All to 10 = Very Enjoyable)?
 Please use a 10-point Likert scale to poll the following responses and take a screenshot of the results.

- Spoke participants overall (average) rating out of 10 __________________________________________________
- Hub Team: Do you measure this at routine milestones? (Yes/No) __________________________________________________
- Hub Team: If yes, have you observed a change over time? (Increase/Decrease/Stable) __________________________________________________
- Hub Team: If you have observed a change, over what period? (months) __________________________________________________
- Hub Team: Do you routinely measure this with another tool, if so, what tool? __________________________________________________
- Hub Team: If you do not collect this data, please explain why __________________________________________________

**15** **Spoke Participant Case Presentations:** For this ECHO Network, please confirm the following metrics:

- What is the total number of individual spoke participants who have presented cases since this Network launched? __________________________________________________
- What is the total number of individual spoke participants who presented cases in the past 12 months? __________________________________________________
- What is the proportion of spoke participants who presented cases versus those who did not in the past 12 months? (percentage) __________________________________________________

**16** **Number of teleECHO sessions that include a case presentation:**For this ECHO Network, please confirm the following metrics:

- What is the proportion of sessions with case presentations from any participant (spoke and/or panel) since launching? (percentage) __________________________________________________
- What is the proportion of sessions with case presentations from any participant (spoke and/or panel) in the 12 months preceding your last session? (percentage) __________________________________________________

**17** **Spoke participant safety and comfort – case presentations:** Administer this question for **Spoke Participants** to complete a **single session poll:**
How would you rate your sense of safety and comfort in volunteering to present cases for discussion in this ECHO Network? (Scale: 0 = Not Very Safe/Comfortable at All to 10 = Very Safe/Comfortable)?
Please use a 10-point Likert scale to poll the following responses and take a screenshot of the results.

- Spoke participants overall (average) rating out of 10 __________________________________________________
- Hub Team: Do you measure this at routine milestones? (Yes/No) __________________________________________________
- Hub Team: If yes, have you observed a change over time? (Increase/Decrease/Stable) __________________________________________________
- Hub Team: If you have observed a change, over what period? (months) __________________________________________________
- Hub Team: Do you routinely measure this with another tool, if so, what tool? __________________________________________________
- Hub Team: If you do not collect this data, please explain why __________________________________________________

**18** **Spoke participant satisfaction – didactic content:** Administer this question for **Spoke Participants** to complete a **single session poll:**
How would you rate your satisfaction with didactic content, panel expert(s) representation/hub team support in this ECHO Network? (Scale: 0 = Not Very Satisfied at All to 10 = Very Satisfied)?
Please use a 10-point Likert scale to poll the following responses and take a screenshot of the results.

- Spoke participants overall (average) rating of didactic content out of 10 __________________________________________________
- Spoke participants overall (average) rating of panel expertise and hub support out of 10 __________________________________________________
- Hub Team: Do you measure this at routine milestones? (Yes/No) __________________________________________________
- Hub Team: If yes, have you observed a change over time? (Increase/Decrease/Stable) __________________________________________________
- Hub Team: If you have observed a change, over what period? (months) __________________________________________________
- Hub Team: Do you routinely measure this with another tool, if so, what tool? __________________________________________________
- Hub Team: If you do not collect this data, please explain why __________________________________________________

**19** **Spoke participant satisfaction – case discussions:** Administer this question for **Spoke Participants** to complete a **single session poll:**
How would you rate your satisfaction with the overall learning/advice/support gained from case presentation and discussion (applies to individual case presenter, as well as other spoke participants learning from the case) and recommendations in this ECHO Network? (Scale: 0 = Not Very Satisfied at All to 10 = Very Satisfied)? Please use a 10-point Likert scale to poll the following responses and take a screenshot of the results.

- Spoke participants overall (average) rating out of 10 __________________________________________________
- Hub Team: Do you measure this at routine milestones? (Yes/No) __________________________________________________
- Hub Team: If yes, have you observed a change over time? (Increase/Decrease/Stable) __________________________________________________
- Hub Team: If you have observed a change, over what period? (months) __________________________________________________
- Hub Team: Do you routinely measure this with another tool, if so, what tool? __________________________________________________
- Hub Team: If you do not collect this data, please explain why __________________________________________________

**20** **Re-presentation of Cases:**For this ECHO Network, please confirm the following metrics:

- What is the proportion of spoke participants who represent a particular case presentation (same patient/consumer/client/scenario) more than once? (percentage) __________________________________________________

**21** **Spoke Participant Satisfaction – In-session dialogue:** Administer this question for **Spoke Participants** to complete a **single session poll:**
How would you rate your satisfaction with the opportunity to contribute to the dialogue, (i.e.: ask questions, make recommendations whether verbally or non-verbally) in this ECHO Network? (Scale: 0 = Not Very Satisfied at All to 10 = Very Satisfied)?
Please use a 10-point Likert scale to poll the following responses and take a screenshot of the results.

- Spoke participants overall (average) rating of opportunity to ask questions out of 10 __________________________________________________
- Spoke participants overall (average) rating of opportunity to make recommendations out of 10 __________________________________________________
- Hub Team: Do you measure this at routine milestones? (Yes/No) __________________________________________________
- Hub Team: If yes, have you observed a change in opportunity to ask questions over time? (Increase/Decrease/Stable) __________________________________________________
- Hub Team: If yes, have you observed a change in opportunity to make recommendations over time? (Increase/Decrease/Stable) __________________________________________________
- Hub Team: If you have observed a change, over what period? (months) __________________________________________________
- Hub Team: Do you routinely measure this with another tool, if so, what tool? __________________________________________________
- Hub Team: If you do not collect this data, please explain why __________________________________________________

**22** **Spoke Participant safety and comfort – attending sessions:** Administer this question for **Spoke Participants** to complete as a **single session poll:**
How would you rate your overall sense of feeling safe, supported, and welcomed in this ECHO Network? (Scale: 0 = Not Very Safe/Supported/Welcomed at All to 10 = Very Safe/Supported/Welcomed)
Please use a 10-point Likert scale to poll the following responses and take a screenshot of the results.

- Spoke participants overall (average) rating of feeling safe out of 10 __________________________________________________
- Spoke participants overall (average) rating of feeling supported out of 10 __________________________________________________
- Spoke participants overall (average) rating of feeling welcomed out of 10 __________________________________________________
- Hub Team: Do you measure this at routine milestones? (Yes/No) __________________________________________________
- Hub Team: If yes, have you observed a change in feeling safe over time? (Increase/Decrease/Stable) __________________________________________________
- Hub Team: If yes, have you observed a change in feeling supported over time? (Increase/Decrease/Stable) __________________________________________________
- Hub Team: If yes, have you observed a change in feeling welcomed over time? (Increase/Decrease/Stable) __________________________________________________
- Hub Team: If you have observed a change, over what period? (months) __________________________________________________
- Hub Team: Do you routinely measure this with another tool, if so, what tool? __________________________________________________
- Hub Team: If you do not collect this data, please explain why __________________________________________________

**23** **Spoke Participant contribution to discussions:**For this ECHO Network, please confirm the following metrics:
Since launching this network, have you measured an increase in the level of spoke participants contributing to the discussion verbally or via chat?

- Hub team: If yes, have you observed a change in spoke participants contributing to the discussion over time? (Increase/Decrease/Stable) __________________________________________________
- What has been the change as a percentage? __________________________________________________
- Hub Team: If you have observed a change, over what period? (months) __________________________________________________
- What tool do you routinely use to measure this? __________________________________________________
- If you do not collect this data, please explain why __________________________________________________

**24** **Spoke Participants inviting colleagues:**For this ECHO Network, please confirm the following metrics:
What is the proportion of case presentations that were co-presented by spoke participants versus single participant presentations?

- What is the proportion of case presentations co-presented by more than one spoke participant? (percentage) __________________________________________________
- If you do not collect this data, please explain why __________________________________________________

**25** **Network Codesign:**For this ECHO Network, please confirm the following metrics:
Which of the below stakeholder groups were consulted during through a co-design process before launch?
Please enter the total number of individual respondents across each relevant stakeholder groups your team consulted with.

- Prospective spoke participants __________________________________________________
- Consumers __________________________________________________
- System managers __________________________________________________
- Subject matter experts/Prospective panelists __________________________________________________
- Others - please specify each group and number of respondents __________________________________________________

**26** **Network alignment to priorities and metrics:** Was the establishment of this ECHO network driven by an intent to align to a government priority/organisational strategy/quality indicator/funding opportunity? Please select all that are relevant to this network.

- Government priorities
- Organisational strategy
- Quality indicator/s
- Funding opportunity
- Other - please specify __________________________________________________
- If none, please explain your intent __________________________________________________

**27** **Learning Needs Assessment:** For this ECHO network, what percentage of priorities/topics/themes that were identified in the Learning Needs Assessment were represented in the didactic curriculum?

- Percentage __________________________________________________

**28** **Interactivity:** Since launching this ECHO network, what has been the increase in the level of interactivity achieved amongst spoke participants AND panelists during sessions (on camera, chat, verbal, non-verbal, volunteering to present cases)?

- Do you currently collect this data? (Yes/No) __________________________________________________
- If you do not collect this data, please explain why not? __________________________________________________
- If yes, has this score increased, decreased or stayed stable over time? __________________________________________________
- If yes, what has been the change as a percentage? __________________________________________________
- If yes, what tool/s do you use - e.g., session recording audits, timestamps, etc.? __________________________________________________
- If yes, how frequently do you measure this? __________________________________________________

**29** **Balance in dialogue:** Administer this question for **Spoke Participants** to complete a **single session poll**:
How would you rate your satisfaction for the balance in dialogue contributed by panelists vs spoke participants in this ECHO Network? (Scale: 0 = Not Very Satisfied at All to 10 = Very Satisfied)
Please use a 10-point Likert scale to poll the following responses and take a screenshot of the results.

- Spoke participants overall (average) rating for balance in dialogue out of 10 __________________________________________________
- Hub Team: Proportion of participants rating 5 and above (percentage) __________________________________________________
- Hub Team: Do you measure this at routine milestones? (Yes/No) __________________________________________________
- Hub Team: If yes, have you observed a change over time? (Increased/Decreased/Stable) __________________________________________________
- Hub Team: If you have observed a change, over what period? (months) __________________________________________________
- Hub Team: Do you routinely measure this with another tool, if so, what tool? __________________________________________________
- Hub Team: If you do not collect this data, please explain why __________________________________________________

**30** **Non-hierarchical Forum:** Administer this question for **Spoke Participants** to complete a **single session poll**:
How would you rate your satisfaction for this ECHO Network's sessions being a non-hierarchical, professional forum for knowledge sharing is fostered by panelists? (Scale: 0 = Not Very Satisfied at All to 10 = Very Satisfied)
Please use a 10-point Likert scale to poll the following responses and take a screenshot of the results.

- Spoke participants overall (average) rating for non-hierarchical and professional forum out of 10 __________________________________________________
- Hub Team: Proportion of participants rating 5 and above (percentage) __________________________________________________
- Hub Team: Do you measure this at routine milestones? (Yes/No) __________________________________________________
- Hub Team: If yes, have you observed a change over time? (Increased/Decreased/Stable) __________________________________________________
- Hub Team: If you have observed a change, over what period? (months) __________________________________________________
- Hub Team: Do you routinely measure this with another tool, if so, what tool? __________________________________________________
- Hub Team: If you do not collect this data, please explain why __________________________________________________

**31** **Fidelity Assurance:** For this ECHO network, have you ever used the Anatomy of an ECHO resource, or teleECHO scorecard as fidelity assurance tools?

- Anatomy of an ECHO
- TeleECHO Scorecard
- Neither tool used
- Other tool - please specify __________________________________________________
- If you do not collect this data, please explain why __________________________________________________

**32** **Pre-Launch Planning:** For this ECHO network, have you documented planning and evaluation processes (Learner Needs Assessment, Implementation Plan, Evaluation Plan) and activities (mock ECHO sessions, panelist/coordinator onboarding, additional Immersion training, etc) prior to launching this ECHO Network? Please select all that are relevant to this network.

- Learner Needs Assessment
- Implementation Plan
- Evaluation Plan
- Mock ECHO sessions
- Panelist onboarding
- Coordinator training
- Additional Immersion training
- If you do not collect this data, please explain why __________________________________________________

**33** **Communication processes:** For this ECHO network, have you established effective communication processes to routinely engage with/promote the network to stakeholders outside of ECHO sessions? Please select all that are relevant to this network.

- Communication and engagement strategy/plan/procedures
- Communication and engagement mailing lists
- Routine iECHO use for up to date participant records
- Localized communication templates for consistent messaging
- Other - please specify __________________________________________________
- If you do not collect this data, please explain why __________________________________________________

**34** **Panelist Experience and Satisfaction:** Administer this question for **Panelists** to complete a **single session poll**:
How would you rate the level of satisfaction with your panelist experience (enjoyment, high value, time efficient) in this ECHO Network? (Scale: 0 = Not Very Satisfied at All to 10 = Very Satisfied)
Please use a 10-point Likert scale to poll the following responses and take a screenshot of the results.

- Panelists overall (average) rating for experience out of 10 __________________________________________________
- Hub Team: Proportion of panelists rating 5 and above (percentage) __________________________________________________
- Hub Team: Do you measure this at routine milestones? (Yes/No) __________________________________________________
- Hub Team: If yes, have you observed a change over time? (Increased/Decreased/Stable) __________________________________________________
- Hub Team: If you have observed a change, over what period? (months) __________________________________________________
- Hub Team: Do you routinely measure this with another tool, if so, what tool? __________________________________________________
- Hub Team: If you do not collect this data, please explain why __________________________________________________

**35** **Network Facilitation:** Administer this question for **Panelists** to complete a **single session poll**:
How would you rate your agreement that your ECHO Network has high levels of strong and organised facilitation role/function, panel cohesion and satisfaction during sessions? (Scale: 0 = Do Not Agree at All to 10 = Very High Agreement)
Please use a 10-point Likert scale to poll the following responses and take a screenshot of the results.

- Panelists overall (average) rating of Strong Facilitation function out of 10 __________________________________________________
- Hub Team: Proportion of panelists rating 5 and above for Strong Facilitation. (percentage) __________________________________________________
- Panelists overall (average) rating of Panel Cohesion out of 10 __________________________________________________
- Hub Team: Proportion of panelists rating 5 and above for Panel Cohesion. (percentage) __________________________________________________
- Panelists overall (average) rating of Session Satisfaction out of 10 __________________________________________________
- Hub Team: Proportion of panelists rating 5 and above for Session Satisfaction. (percentage) __________________________________________________
- Hub Team: Do you measure this at routine milestones? (Yes/No) __________________________________________________
- Hub Team: If yes, have you observed a change over time? (Increased/Decreased/Stable) __________________________________________________
- Hub Team: If you have observed a change, over what period? (months) __________________________________________________
- Hub Team: Do you routinely measure this with another tool, if so, what tool? __________________________________________________
- Hub Team: If you do not collect this data, please explain why __________________________________________________

**36** **Network Panelist Recruitment and Retention:** Administer this question for **Panelists** to complete a **single session poll**:
How would you rate your agreement that your ECHO Network has demonstrated ability to recruit and retain Champion, Facilitator, Panelists with qualifications, skills, expertise, experience, that presents well and makes spokes feel comfortable? (Scale: 0 = Do Not Agree at All to 10 = Very High Agreement)
Please use a 10-point Likert scale to poll the following responses and take a screenshot of the results.

- Panelists overall (average) rating of Champion Recruitment & Retention out of 10 __________________________________________________
- Hub Team: Proportion of panelists rating 5 and above for Champion Recruitment & Retention. (percentage) __________________________________________________
- Panelists overall (average) rating of Panel Member Recruitment & Retention out of 10 __________________________________________________
- Hub Team: Proportion of panelists rating 5 and above for Panel Member Recruitment and Retention. (percentage) __________________________________________________
- Hub Team: Do you measure this at routine milestones? (Yes/No) __________________________________________________
- Hub Team: If yes, have you observed a change over time? (Increased/Decreased/Stable) __________________________________________________
- Hub Team: If you have observed a change, over what period? (months) __________________________________________________
- Hub Team: Do you routinely measure this with another tool, if so, what tool? __________________________________________________
- Hub Team: If you do not collect this data, please explain why __________________________________________________

**37** **Network Panelist Satisfaction - Case Discussions:** Administer this question for **Panelists** to complete a **single session poll**:
How would you rate your agreement that your ECHO Network has demonstrated high levels of panel satisfaction with the case-based learning component of sessions? (Scale: 0 = Very Strong Disagreement to 10 = Very Strong Agreement)
Please use a 10-point Likert scale to poll the following responses and take a screenshot of the results.

- Panelists satisfaction overall (average) rating of Case Discussions out of 10 __________________________________________________
- Hub Team: Proportion of panelists rating 5 and above for Case Discussions. (percentage) __________________________________________________
- Hub Team: Do you measure this at routine milestones? (Yes/No) __________________________________________________
- Hub Team: If yes, have you observed a change over time? (Increased/Decreased/Stable) __________________________________________________
- Hub Team: If you have observed a change, over what period? (months) __________________________________________________
- Hub Team: Do you routinely measure this with another tool, if so, what tool? __________________________________________________
- Hub Team: If you do not collect this data, please explain why __________________________________________________

**38** **Spoke Participant Confidence:** Administer this question for **Spoke Participants** to complete a **single session poll**:
Please rate your current level of **confidence** in managing cases locally, as impacted by your participation in this ECHO Network. (Scale: 0 = Very Low to 10 = Very High)
Please use a 10-point Likert scale to poll the following responses and take a screenshot of the results.

- Participants' overall (average) rating of confidence out of 10 __________________________________________________
- Hub Team: Proportion of participants rating 5 and above for confidence. (percentage) __________________________________________________
- Hub Team: Do you measure this at routine milestones? (Yes/No) __________________________________________________
- Hub Team: If yes, have you observed a change over time? (Increased/Decreased/Stable) __________________________________________________
- Hub Team: If you have observed a change, over what period? (months) __________________________________________________
- Hub Team: If Yes, what has been the change observed over time? (percentage) __________________________________________________
- Hub Team: Do you routinely measure this with another tool, if so, what tool? __________________________________________________
- Hub Team: If you do not collect this data, please explain why __________________________________________________

**39** **Spoke Participant Competence:** Administer this question for **Spoke Participants** to complete a **single session poll**:
Please rate your current level of **competence** in managing cases locally, as impacted by your participation in this ECHO Network. (Scale: 0 = Very Low to 10 = Very High)
Please use a 10-point Likert scale to poll the following responses and take a screenshot of the results.

- Participants' overall (average) rating of competence out of 10 __________________________________________________
- Hub Team: Proportion of participants rating 5 and above for competence. (percentage) __________________________________________________
- Hub Team: Do you measure this at routine milestones? (Yes/No) __________________________________________________
- Hub Team: If yes, have you observed a change over time? (Increased/Decreased/Stable) __________________________________________________
- Hub Team: If you have observed a change, over what period? (months) __________________________________________________
- Hub Team: If Yes, what has been the change observed over time? (percentage) __________________________________________________
- Hub Team: Do you routinely measure this with another tool, if so, what tool? __________________________________________________
- Hub Team: If you do not collect this data, please explain why __________________________________________________

**40** **Spoke Participant Knowledge/Skills:** Administer this question for **Spoke Participants** to complete a **single session poll**:
Please rate your current level of **knowledge/skills** to manage cases locally, as impacted by your participation in this ECHO Network. (Scale: 0 = Very Low to 10 = Very High)
Please use a 10-point Likert scale to poll the following responses and take a screenshot of the results.

- Participants' overall (average) rating of knowledge/skills out of 10 __________________________________________________
- Hub Team: Proportion of participants rating 5 and above for knowledge/skills. (percentage) __________________________________________________
- Hub Team: Do you measure this at routine milestones? (Yes/No) __________________________________________________
- Hub Team: If yes, have you observed a change over time? (Increased/Decreased/Stable) __________________________________________________
- Hub Team: If you have observed a change, over what period? (months) __________________________________________________
- Hub Team: If Yes, what has been the change observed over time? (percentage) __________________________________________________
- Hub Team: Do you routinely measure this with another tool, if so, what tool? __________________________________________________
- Hub Team: If you do not collect this data, please explain why __________________________________________________

**41** **Spoke Participant Capacity:** Administer this question for **Spoke Participants** to complete a **single session poll**:
Please rate your current level of **capacity** to manage more cases locally (i.e., has your caseload capacity increased?), as impacted by your participation in this ECHO Network (Scale: 0 = Very Low to 10 = Very High)
Please use a 10-point Likert scale to poll the following responses and take a screenshot of the results.

- Participants' overall (average) rating of capacity out of 10 __________________________________________________
- Hub Team: Proportion of participants rating 5 and above for capacity. (percentage) __________________________________________________
- Hub Team: Do you measure this at routine milestones? (Yes/No) __________________________________________________
- Hub Team: If yes, have you observed a change over time? (Increased/Decreased/Stable) __________________________________________________
- Hub Team: If you have observed a change, over what period? (months) __________________________________________________
- Hub Team: If Yes, what has been the change observed over time? (percentage) __________________________________________________
- Hub Team: Do you routinely measure this with another tool, if so, what tool? __________________________________________________
- Hub Team: If you do not collect this data, please explain why __________________________________________________

**42** **Spoke Participants becoming local experts:** Administer this question for **Spoke Participants** to complete a **single session poll**:
As a result of participating in this ECHO Network, please rate your perceived level of change in **becoming a local expert** to whom colleagues refer to and collaborate with for support on cases (Scale: 0 = Very Low to 10 = Very High)
Please use a 10-point Likert scale to poll the following responses and take a screenshot of the results.

- Participants' overall (average) rating of perceived level of change in becoming a local expert out of 10 __________________________________________________
- Hub Team: Proportion of participants rating 5 and above for perceived level of change in becoming a local expert. (percentage) __________________________________________________
- Hub Team: Do you measure this at routine milestones? (Yes/No) __________________________________________________
- Hub Team: If yes, have you observed a change over time? (Increased/Decreased/Stable) __________________________________________________
- Hub Team: If you have observed a change, over what period? (months) __________________________________________________
- Hub Team: If Yes, what has been the change observed over time? (percentage) __________________________________________________
- Hub Team: Do you routinely measure this with another tool, if so, what tool? __________________________________________________
- Hub Team: If you do not collect this data, please explain why __________________________________________________

**43** **Spoke Participants change in practice:** Administer this question for **Spoke Participants** to complete a **single session poll**:
Following your participation in this ECHO network, have you applied at least one **change in your practice**? Please select as many practice changes from the below list as relevant to you. 
**Hub team**: take a screenshot of the results and enter the totals for each item in the list below.

- No changes in practice __________________________________________________
- Changes in information collected at referral/intake/assessment __________________________________________________
- Changes in Method/Approach/Process to assessing patient/client/consumer/student __________________________________________________
- Changes in Techniques to work with patient/client/consumer/student __________________________________________________
- Changes in Program/Service Referral Processes __________________________________________________
- Changes in Organisational procedures __________________________________________________
- Changes in Program/Service overall __________________________________________________
- Changes in Staffing __________________________________________________
- Other - please specify __________________________________________________
- Hub Team: Proportion of participants reporting at least one change. (percentage) __________________________________________________
- Hub Team: Do you measure this at routine milestones? (Yes/No) __________________________________________________
- Hub Team: If Yes, what has been the change observed over time? (percentage) __________________________________________________
- Hub Team: If you have observed a change, over what period? (months) __________________________________________________
- Hub Team: Do you routinely measure this with another tool, if so, what tool? __________________________________________________
- Hub Team: If you do not collect this data, please explain why. __________________________________________________

**44** **Spoke Participant Self-Efficacy:** Administer this question for **Spoke Participants** to complete a **single session poll**:
Please rate your current level of professional **self-efficacy**, as impacted by your participation in this ECHO Network (Scale: 0 = Very Low to 10 = Very High)
Please use a 10-point Likert scale to poll the following responses and take a screenshot of the results.

- Participants' overall (average) rating of self-efficacy out of 10 __________________________________________________
- Hub Team: Proportion of participants rating 5 and above for self-efficacy. (percentage) __________________________________________________
- Hub Team: Do you measure this at routine milestones? (Yes/No) __________________________________________________
- Hub Team: If yes, have you observed a change over time? (Increased/Decreased/Stable) __________________________________________________
- Hub Team: If you have observed a change, over what period? (months) __________________________________________________
- Hub Team: If Yes, what has been the change observed over time? (percentage) __________________________________________________
- Hub Team: Do you routinely measure this with another tool, if so, what tool? __________________________________________________
- Hub Team: If you do not collect this data, please explain why __________________________________________________

**45** **Spoke Participant Professional Isolation:** Administer this question for **Spoke Participants** to complete a **single session poll**:
Please rate your current level of **professional isolation** (i.e.: has this sense of isolation been reduced?), as impacted by your participation in this ECHO Network (Scale: 0 = Very Low to 10 = Very High)
Please use a 10-point Likert scale to poll the following responses and take a screenshot of the results.

- Participants' overall (average) rating of professional isolation out of 10 __________________________________________________
- Hub Team: Proportion of participants rating 5 and above for professional isolation. (percentage) __________________________________________________
- Hub Team: Do you measure this at routine milestones? (Yes/No) __________________________________________________
- Hub Team: If yes, have you observed a change over time? (Increased/Decreased/Stable) __________________________________________________
- Hub Team: If you have observed a change, over what period? (months) __________________________________________________
- Hub Team: If Yes, what has been the change observed over time? (percentage) __________________________________________________
- Hub Team: Do you routinely measure this with another tool, if so, what tool? __________________________________________________
- Hub Team: If you do not collect this data, please explain why __________________________________________________

**46** **Spoke Participant Joy of Work:** Administer this question for **Spoke Participants** to complete a **single session poll**:
Please rate your current level of **joy of work**, (i.e.: has this joy of work been increased?) as impacted by your participation in this ECHO Network. (Scale: 0 = Very Low to 10 = Very High)
Please use a 10-point Likert scale to poll the following responses and take a screenshot of the results.

- Participants' overall (average) rating of joy of work out of 10 __________________________________________________
- Hub Team: Proportion of participants rating 5 and above for joy of work. (percentage) __________________________________________________
- Hub Team: Do you measure this at routine milestones? (Yes/No) __________________________________________________
- Hub Team: If yes, have you observed a change over time? (Increased/Decreased/Stable) __________________________________________________
- Hub Team: If you have observed a change, over what period? (months) __________________________________________________
- Hub Team: If Yes, what has been the change observed over time? (percentage) __________________________________________________
- Hub Team: Do you routinely measure this with another tool, if so, what tool? __________________________________________________
- Hub Team: If you do not collect this data, please explain why __________________________________________________

**47** **Spoke Participant Knowledge-sharing relationships:** Administer this question for **Spoke Participants** to complete a **single session poll**:
Please rate your current **strength of relationships with local colleagues** with which you **share knowledge**, as impacted by your participation in this ECHO Network (i.e. have these relationships been strengthened?). (Scale: 0 = Very Low to 10 = Very High)
Please use a 10-point Likert scale to poll the following responses and take a screenshot of the results.

- Participants' overall (average) rating of knowledge-sharing relationships out of 10 __________________________________________________
- Hub Team: Proportion of participants rating 5 and above for knowledge-sharing relationships. (percentage) __________________________________________________
- Hub Team: Do you measure this at routine milestones? (Yes/No) __________________________________________________
- Hub Team: If yes, have you observed a change over time? (Increased/Decreased/Stable) __________________________________________________
- Hub Team: If you have observed a change, over what period? (months) __________________________________________________
- Hub Team: If Yes, what has been the change observed over time? (percentage) __________________________________________________
- Hub Team: Do you routinely measure this with another tool, if so, what tool? __________________________________________________
- Hub Team: If you do not collect this data, please explain why __________________________________________________

**48** **Spoke Participant Satisfaction - Service Improvements:** Administer this question for **Spoke Participants** to complete a **single session poll**:
Please rate your current level **satisfaction with improvements** observed in service utilization, service wait times, distance travelled to access services by patients/consumers/clients, as impacted by your participation in this ECHO Network (Scale: 0 = Very Low to 10 = Very High)
Please use a 10-point Likert scale to poll the following responses and take a screenshot of the results.

- Participants' overall (average) rating of service improvements out of 10 __________________________________________________
- Hub Team: Proportion of participants rating 5 and above for service improvements. (percentage) __________________________________________________
- Hub Team: Do you measure this at routine milestones? (Yes/No) __________________________________________________
- Hub Team: If yes, have you observed a change over time? (Increased/Decreased/Stable) __________________________________________________
- Hub Team: If you have observed a change, over what period? (months) __________________________________________________
- Hub Team: If Yes, what has been the change observed over time? (percentage) __________________________________________________
- Hub Team: Do you routinely measure this with another tool, if so, what tool? __________________________________________________
- Hub Team: If you do not collect this data, please explain why __________________________________________________

**49** **Hub Team:** Do you feel that the success indicators utilised in this study are applicable and exhaustive?

- Yes/No __________________________________________________
- Please provide any feedback regarding these success indicators. __________________________________________________

Thank you
We appreciate the time you have taken to participate. Please click the arrow in the below right corner to complete your Part B single submission.
 **You can proceed to recomplete Part B for all of your additional ECHO Networks by copying this link into your browser:**
 **** Qualtrics Link for Part B submission embedded here ****

If you have any questions, please contact [Perrin.Moss@health.qld.gov.au](mailto:Perrin.Moss@health.qld.gov.au) so a Zoom information session can be scheduled at a convenient time for you.


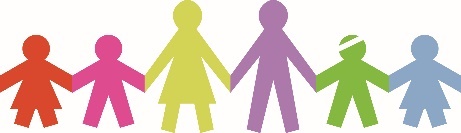

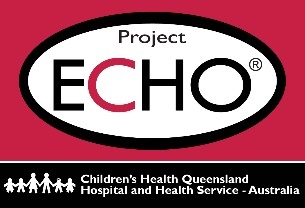

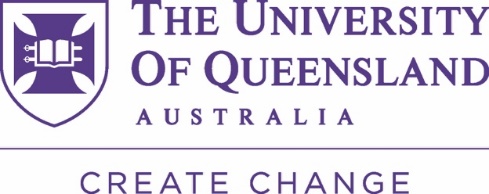


**Addendum: Polling survey questions excerpt for Part A and Part B:**

**Poll questions:**

To support participating teams ease of inputting the Participant Guide polling questions into Zoom, the research team have issued this document to enable the copying and pasting of question content into Zoom.

Please note this addendum only covers the polling specific questions from the overarching Participant Guide.

The remaining questions can still be reported on directly by the hub team via the Qualtrics link after the polls have been completed for Part B for each ECHO Network your hub is submitting data for.

Please copy and paste the text from these polls into the Zoom polling function prior to your next ECHO Network session to administer these questions to your network members. You can then export the results following the session for entering into the Qualtrics survey links along with your responses for the other questions included in the Participant Guide for Part A and Part B.

If your ECHO Network is currently paused for seasonal breaks, you can also input these polls into your preferred survey platform (REDCap, Survey Monkey, other) to collect responses from your Network members, and input the results into the Qualtrics links below as outlined for each question in Part A and Part B.

**Part A:** Once off submission per ECHO Hub

| **Poll #** | **Study Guide Part A Question Reference for Qualtrics data submission** |
| --- | --- |
| 1 | Q29: **Hub teams:** How would you rate your ECHO hub team’s understanding of the ECHO model, its theoretical and practical application, and potential benefits in your context?  (0 = Very Low to 10 = Very High Likert Scale) |

**Part B:** Repeat these questions for **EACH** ECHO Network your hub is submitting data for

| **Poll #** | **Study Guide Part B Question Reference for Qualtrics data submission** |
| --- | --- |
| 1 | Q14: **Spoke participants:** How would you rate your experience as a spoke participant in this ECHO Network?  (0 = Not Very Enjoyable At All to 10 = Very Enjoyable Likert Scale) |
| 2 | Q17: **Spoke participants:** How would you rate your sense of safety and comfort in volunteering to present cases for discussion in this ECHO Network?  (0 = Not Very Safe/Comfortable at All to 10 = Very Safe/Comfortable Likert Scale) |
| 3 | Q18: **Spoke participants:** How would you rate your satisfaction with didactic content, panel expert(s) representation/hub team support in this ECHO Network?  (0 = Not Very Satisfied At All to 10 = Very Satisfied Likert Scale) |
| 4 | Q19: **Spoke participants:** How would you rate your satisfaction with the overall learning/advice/support gained from case presentation and discussion and recommendations in this ECHO Network?  (0 = Not Very Satisfied At All to 10 = Very Satisfied Likert Scale) |
| 5 | Q21: **Spoke participants:** How would you rate your satisfaction with the opportunity to contribute to dialogue, (i.e.: ask questions, make recommendations whether verbally or non-verbally) in this ECHO Network?  (0 = Not Very Satisfied At All to 10 = Very Satisfied Likert Scale) |
| 6 | Q22: **Spoke participants:** How would you rate your overall sense of feeling safe, supported, and welcomed in this ECHO Network?  (0 = Not Very Safe/Supported/Welcomed At All to 10 = Very Safe/Supported/Welcomed Likert Scale) |
| 7 | Q29: **Spoke participants:** How would you rate your satisfaction for the balance of dialogue contributed by panellists versus spoke participants in this ECHO Network?  (0 = Not Very Satisfied At All to 10 = Very Satisfied Likert Scale) |
| 8 | Q30: **Spoke participants:** How would you rate your satisfaction for this ECHO Network’s sessions being a non-hierarchical, professional forum for knowledge sharing is fostered by panellists?  (0 = Not Very Satisfied At All to 10 = Very Satisfied Likert Scale) |
| 9 | Q34: **Spoke participants:** How would you rate the level of satisfaction with your panellist experience (enjoyable, high value, time efficient) for this ECHO Network?  (0 = Not Very Satisfied At All to 10 = Very Satisfied Likert Scale) |
| 10 | Q35: **Panelists:** How would you rate your agreement that your ECHO Network has high levels of strong and organised facilitation role/function, panel cohesion and satisfaction during sessions?  (0 = Do Not Agree At All to 10 = Very High Agreement Likert Scale) |
| 11 | Q36: **Panelists:** How would you rate your agreement that your ECHO Network has demonstrated ability to recruit & retain Champion, Facilitator, Panellists with qualifications, skills, expertise, experience, that presents well & makes spokes feel comfortable?  (0 = Do Not Agree At All to 10 = Very High Agreement Likert Scale) |
| 12 | Q37: **Panelists:** How would you rate your agreement that your ECHO Network has demonstrated high levels of panel satisfaction with the case-based learning component of sessions?  (0 = Very Strong Disagreement to 10 = Very Strong Agreement Likert Scale) |
| 13 | Q38: **Spoke participants:** Please rate your current level of confidence in managing cases locally, as impacted by your participation in this ECHO Network.  (0 = Very Low to 10 = Very High Likert Scale) |
| 14 | Q39: **Spoke participants:** Please rate your current level of competence in managing cases locally, as impacted by your participation in this ECHO Network.  (0 = Very Low to 10 = Very High Likert Scale) |
| 15 | Q40: **Spoke participants:** Please rate your current level of knowledge/skills to manage cases locally, as impacted by your participation in this ECHO Network.  (0 = Very Low to 10 = Very High Likert Scale) |
| 16 | Q41: **Spoke participants:** Please rate your current level of capacity to manage more cases locally (i.e.: has your caseload capacity increased?), as impacted by your participation in this ECHO Network.  (0 = Very Low to 10 = Very High Likert Scale) |
| 17 | Q42: **Spoke participants:** As a result of participating in this ECHO Network, please rate your perceived level of change in becoming a local expert to whom colleagues refer to and collaborate with for support on cases.  (0 = Very Low to 10 = Very High Likert Scale) |
| 18 | Q43: **Spoke participants:** Following your participation in this ECHO Network, have you applied at least one change in your practice? Please select as many practice changes from the below list as relevant to you:   - No change(s) in practice - Changes in information collected at referral/intake/assessment - Changes in Method/Approach/Process to assessing patient/client/consumer/student - Changes in techniques to work with patient/client/consumer/student - Changes in program/service referral processes - Changes in organisational procedures - Changes in program/service overall - Changes in staffing - Other – please specify (by using chat function) |
| 19 | Q44: **Spoke participants:** Please rate your current level of professional self-efficacy, as impacted by your participation in this ECHO Network.  (0 = Very Low to 10 = Very High Likert Scale) |
| 20 | Q45: **Spoke participants:** Please rate your current level of professional isolation (i.e.: has this sense of isolation been reduced?), as impacted by your participation in this ECHO Network?  (0 = Very Low to 10 = Very High Likert Scale) |
| 21 | Q46: **Spoke participants:** Please rate your current level of joy of work, (i.e.: has this joy of work been increased?) as impacted by your participation in this ECHO Network?  (0 = Very Low to 10 = Very High Likert Scale) |
| 22 | Q47: **Spoke participants:** Please rate your current strength of relationships with local colleagues with which you share knowledge, as impacted by your participation in this ECHO Network (i.e.: have these relationships been strengthened?).  (0 = Very Low to 10 = Very High Likert Scale) |
| 23 | Q48: **Spoke participants:** Please rate your current satisfaction with improvements observed in service utilisation, service wait times, distance travelled to access services by patients/consumers/clients, as impacted by your participation in this ECHO Network.  (0 = Very Low to 10 = Very High Likert Scale) |
